# A specific tRNA half, 5’tiRNA-His-GTG, responds to hypoxia via the HIF1α/ANG axis and promotes colorectal cancer progression by regulating LATS2

**DOI:** 10.1186/s13046-021-01836-7

**Published:** 2021-02-15

**Authors:** En-Wei Tao, Hao-Lian Wang, Wing Yin Cheng, Qian-Qian Liu, Ying-Xuan Chen, Qin-Yan Gao

**Affiliations:** 1 Division of Gastroenterology and Hepatology, Shanghai Institute of Digestive Disease, State Key Laboratory for Oncogenes and Related Genes, Key Laboratory of Gastroenterology & Hepatology, Ministry of Health, Ren-Ji Hospital, Shanghai Jiao-Tong University School of Medicine, Renji Hospital, 145 Middle Shandong Road, 200001 Shanghai, China; 2 Institute of Digestive Disease and Department of Medicine and Therapeutics, State Key Laboratory of Digestive Disease, Li Ka Shing Institute of Health Sciences, CUHK Shenzhen Research Institute, The Chinese University of Hong Kong, Hong Kong, 999077 China

**Keywords:** Colorectal cancer, Hypoxia, LATS2, Hippo signaling pathway, tRNA half

## Abstract

**Background:**

Currently, tRNA-derived small RNAs (tsRNAs) are recognized as a novel and potential type of non-coding RNAs (ncRNAs), which participate in various cellular processes and play an essential role in cancer progression. However, tsRNAs involvement in colorectal cancer (CRC) progression remains unclear.

**Methods:**

Sequencing analyses were performed to explore the tsRNAs with differential expression in CRC. Gain- and loss-of functions of 5’tiRNA-His-GTG were performed in CRC cells and xenograft tumor to discover its role in the progression of CRC. Hypoxia culture and hypoxia inducible factor 1 subunit alpha (HIF1α) inhibitors were performed to uncover the biogenesis of 5’tiRNA-His-GTG. The regulation of 5’tiRNA-His-GTG for large tumor suppressor kinase 2 (LATS2) were identified by luciferase reporter assay, western blot, and rescue experiments.

**Results:**

Here, our study uncovered the profile of tsRNAs in human CRC tissues and confirmed a specific tRNA half, 5’tiRNA-His-GTG, is upregulated in CRC tissues. Then, in vitro and in vivo experiments revealed the oncogenic role of 5’tiRNA-His-GTG in CRC and found that targeting 5’tiRNA-His-GTG can induce cell apoptosis. Mechanistically, the generation of 5’tiRNA-His-GTG seems to be a responsive process of tumor hypoxic microenvironment, and it is regulated via the HIF1α/angiogenin (ANG) axis. Remarkably, LATS2 was found to be an important and major target of 5’tiRNA-His-GTG, which renders 5’tiRNA-His-GTG to “turn off” hippo signaling pathway and finally promotes the expression of pro-proliferation and anti-apoptosis related genes.

**Conclusions:**

In summary, the findings revealed a specific 5’tiRNA-His-GTG-engaged pathway in CRC progression and provided clues to design a novel therapeutic target in CRC.

**Supplementary Information:**

The online version contains supplementary material available at 10.1186/s13046-021-01836-7.

## Highlights

1. 5’tiRNA-His-GTG is a novel small non-coding RNA that promotes CRC progression
2. 5’tiRNA-His-GTG is mainly located in cytoplasm and regulated via hypoxia/HIF1α/ANG axis
3. 5’tiRNA-His-GTG inhibits the translation of LATS2 and suppresses the hippo signaling pathway
4. Suggested a new mechanism of 5’tiRNA-His-GTG-engaged pathway in CRC progression

## Background

Colorectal cancer (CRC) is one of the most common cancers and is the second leading cause of cancer-related death globally [1, 2]. Currently, the treatment options for CRC are mainly surgical resection and chemotherapy. Research efforts in recent decades have led to reductions in the incidence and mortality of CRC in adults over the age of 50 [3]. However, the low survival rate of patients with advanced CRC indicates that more effective treatments are required [4, 5]. In this regard, the pathological mechanisms driving CRC should be thoroughly investigated to identify novel biomarkers or therapeutic targets.

For decades, non-coding RNAs (ncRNAs) have been shown to play critical roles in both cellular function and human disease. The emerging roles of ncRNAs in cancer development have also been well discovered. Some ncRNAs are stable and detectable in various human bio-fluids, which can be regarded as potential non-invasive biomarkers in cancers [6]. Advances in oligonucleotide drug delivery make it more available to target ncRNAs against malignant tumors [7].

To date, an explosion of studies have focused on microRNAs (miRNAs), long non-coding RNAs (lncRNAs), piwi-interacting RNA (piRNAs), and circular RNAs (circRNAs), however, researchers rarely concerned tRNA, which is an ancient and well-known adapter that carries amino acids to the ribosome. Intriguingly, recent studies have identified tRNA can be cleaved under stress conditions [8–10], and next-generational sequencing indicated that the cleaved tRNA products are not randomly degraded fragments [11], which implies the biological function of these fragments. In subsequent studies, these fragments derived from tRNA were found to be implicated in various cellular processes, including stress response, ribosome biogenesis, epigenetic regulation, translational inhibition, mRNA stability repression [12–14]. Thus, these fragments were gradually recognized as a novel class of small non-coding RNAs, named tRNA-derived small RNAs (tsRNAs). tsRNAs are classified into two major types: tRNA halves (30–45 nucleotides) and smaller tRNA fragments (tRFs) (14–30 nucleotides), based on their enzyme cleavage site and the length [15]. tRNA halves, also called tRNA-derived and stress-induced small RNAs (tiRNAs), are induced by stressors such as hypoxia, amino acid deficiency, UV radiation, heat shock, oxidative damage, and viral infection [14]. Regarding their specific biogenesis, current studies suggest that mature tRNAs are cleaved by angiogenin (ANG) in the anticodon-loop region, generating 5’tiRNAs (30–35 nucleotides) and 3’tiRNAs (40–45 nucleotides) [8, 9, 16]. As for tRFs, they have three subclasses: 5’tRFs, 3’tRFs, i-tRFs. However, the biogenesis mechanism of tRFs remains unknown, it may be related to Dicer cleavage in D-loop or T-loop [17–19].

Both subtypes of tsRNA are closely related to the development of tumors [14, 20, 21]. For example, specific tRFs derived from tRNA-Glu, tRNA-Asp, tRNA-Gly, and tRNA-Tyr suppress breast cancer by binding to Y box binding protein 1 (YBX1) and destabilizing oncogenic transcripts [22]. One specific tRF, derived from tRNA-Leu, was identified as a regulator of ribosome biogenesis and is a potential target to treat cancer [23]. Besides, researchers also revealed that the sex hormone signaling pathway promotes angiogenin-mediated cleavage of mature tRNA and generates several 5’tiRNAs to enhance cell proliferation of breast and prostate cancers [24]. More recently, a novel tRNA half named 5’tiRNA-Val was found to suppress breast cancer cell’s proliferation, migration, and invasion via inhibiting the FZD3/Wnt/β-Catenin signaling pathway [25].

Despite the emerging interest in tsRNAs, the biogenesis and mechanism of tsRNAs remain largely unknown. In the present study, we identified that 5’tiRNA-His-GTG plays an oncogenic role in CRC progression. Regulated by the hypoxia inducible factor 1 subunit alpha (HIF1α)/ANG axis, 5’tiRNA-His-GTG interacts with large tumor suppressor kinase 2 (LATS2) to suppress hippo signaling. Strategies to disrupt the 5’tiRNA-His-GTG-engaged pathway might be developed for treatment of colorectal cancer.

## Materials and methods

### CRC samples

We obtained 25 paired CRC tissues and adjacent normal tissues from Ren-ji Hospital, Shanghai Jiao-Tong University School of Medicine (Shanghai, China). Patients were diagnosed clinically and pathologically with colorectal cancer (the clinical information for the patients is listed in Table S1). None of the patients had received radiotherapy or chemotherapy before surgery. Written informed consent was obtained from each study patient. The study protocol was approved by the ethics committee of Shanghai Jiao-Tong University School of Medicine (Shanghai, China). All the research was carried out in accordance with the provisions of the declaration of Helsinki of 1975.

### tsRNA sequence processing and differential expression analysis

RNA samples were extracted from four paired CRC tissues and adjacent normal tissues. The integrity and quantity of RNA were checked before sequencing. RNA modification interferes with small RNA-seq library construction; therefore, an RNA Pretreatment Kit (#: AS-FS-005, Arraystar, USA) was used to remove 3′-aminoacyl, 3′-cP, phosphorylate 5′-OH, and demethylate m1A, m1G, and m3C to promote efficient cDNA reverse transcription. After sequentially ligating 3′ and 5′ small RNA adapters (#: AS-FS-003, Arraystar), cDNA was synthesized and amplified using Illumina’s proprietary reverse transcription (RT) primers and amplification primers. Subsequently, PCR amplified fragments of ~ 135–160 bp were extracted and purified from the polyacrylamide gel and used for library construction. Finally, the completed libraries were quantified using Agilent 2100 Bioanalyzer (Agilent, USA). The libraries were denatured and diluted to a loading volume of 1.3 mL and loaded at a concentration of 1.8 pM onto a reagent cartridge sequenced on an Illumina NextSeq 500 system using a NextSeq 500/550 V2 kit (#FC-404-2005, Illumina, USA), according to the manufacturer’s instructions. Illumina NextSeq 500 raw sequencing read data that passed the Illumina chastity filter were used for subsequent analysis. Trimmed reads (with 5′, 3′-adaptor bases removed) were aligned to mature-tRNA and pre-tRNA reference sequences. Statistical analysis of the alignment results was applied to retain the valid sequences for subsequent tRF & tiRNA expression profiling and differential expression analysis. The RNA sequence data have been deposited in the NCBI Gene Expression Omnibus (GEO) database and are accessible through the GEO Series accession number GSE140327.

### Cell culture

Human colorectal cancer cell lines HCT116, LoVo, RKO, SW1116, Caco2, SW480, and DLD1 were purchased from American Type Culture Collection (ATCC). These cell lines were tested for mycoplasma contamination before use to ensure that they were mycoplasma-free. The cells were maintained in Roswell Park Memorial Institute (RPMI) 1640 or Dulbecco’s modified Eagle’s medium (DMEM) medium supplemented with 10% fetal bovine serum (Gibco, USA). To induce hypoxia, cells were incubated with 1% O_2_, 5% CO_2_, and 94% N_2_ in a hypoxia chamber (YCP-50s; Huaxi Electronic Technologies, Changsha, China).

### RNA extraction and quantitative real-time reverse transcription PCR

Total RNA was extracted from cells and tissues using the Trizol reagent (Life Technologies, USA). A Cytoplasmic and Nuclear RNA Purification Kit (Norgen, Canada) was used to separate RNA from the cytoplasm and nucleus. RNA Pretreatment Kit (#: AS-FS-005, Arraystar, USA) and rtStar™ First-Strand cDNA Synthesis Kit (#: AS-FS-003, Arraystar, USA) were used to build specific cDNA libraries for the quantification of tsRNA in tissues. mRNA and 5’tiRNA-His-GTG were reverse transcribed to cDNA using a PrimeScript RT Reagent Kit (Perfect Real-Time, Takara, Japan) and a Bulge-Loop miRNA qRT-PCR Starter Kit (Ribobio, Guangzhou, China) in cells, respectively. Mature tRNA-His-GTG was reverse transcribed to cDNA using rtStar™ tRNA-optimized First-Strand cDNA Synthesis Kit (#: AS-FS-004, Arraystar). Then, qPCR was performed with SYBR Premix Ex Taq (Takara). The expression levels of tsRNAs and tRNA-His-GTG were normalized to that of U6, and the expression levels of mRNAs were normalized to that of β-actin. The primer used for detecting tRNA-His-GTG was purchased from Arraystar (#: AS-NR-001H-1-074), and the details of the other primers are shown in Table S2.

### Cell transfection

Cells were seeded into plates overnight before transfection. The synthetic single-strand mimic (5’tiRNA-His-GTG mimic, modified with 2′-O-Methyl (2′-O-Me), 50 nM), inhibitor (5’tiRNA-His-GTG inhibitor, modified with 2′-O-Me, 100 nM), and small interfering RNAs (siRNAs, 50 nM) against human *ANG*, *LATS2*, *AGO1*, *AGO2*, *AGO3*, and *AGO4* were purchased from Genepharma Technology (Shanghai, China). The transient transfection of RNA oligonucleotides was performed using the DharmaFECT 1 siRNA transfection reagent (Thermo Scientific Dharmacon Inc., USA). All the plasmids were obtained from Generay Biotechnology (Shanghai, China) and were transfected into CRC cells using the FuGENE transfection reagent (Life Technologies, USA). The sequences of siRNAs and the details of the RNA oligonucleotides are listed in Table S3 and Table S4.

### Cell proliferation, colony formation, and apoptosis assays

Cell proliferation was determined by using a Cell Counting Kit 8 (CCK8) (Dojindo, Japan) according to the manufacturer’s instructions. Treated cells were seeded in 96-well plates at an initial density of 1500 cells (HCT116) or 2000 cells (LoVo, RKO) per well. For the colony formation assay, transfected cells (750–1000 cells per well) were cultured in 6-well plates for 8–10 days, and then fixed with 4% formaldehyde and stained using 0.1% crystal violet. The relative colony formation ability was determined using ImageJ (NIH, USA). The cell apoptosis assay was performed by using an Annexin V-fluorescein isothiocyanate (FITC) Apoptosis Detection Kit I (BD Biosciences, USA). Terminal deoxynulceotidyl transferase nick-end-labeling (TUNEL) staining of tissue sections, used a TUNEL staining Kit (Keygen Biotech, Nanjing, China) according to the manufacturer’s instructions.

### Western blotting and chemical reagents

Western blotting analysis was performed using standard procedures. An anti-β-actin antibody (KC-5A08, Kang Cheng, China) was used as a reference control. The following antibodies were obtained from the indicated sources: Anti-HIF-1α (#: 36169), anti-cleaved-PARP (#: 5625), anti-cleaved-Caspase9 (#: 9505), anti-PARP (#: 9532), anti-Caspase9 (#: 9502), anti-LATS2 (#: 5888), anti-Phospho-YAP-S127 (#: 13008), and anti-YAP (#: 14074) were obtained from Cell Signaling Technology (USA). Anti-ANG (#: ab10600) was obtained from Abcam (UK). The HIF-1α inhibitors LW6 (#: CSN20474) and 2-ME (#: CSN19253) were purchased from CSNpharm (USA).

### Luciferase assay

Dual-luciferase reporter assays were used to evaluate the direct binding between HIF-1α and the *ANG* promoter region, as well as the 3′ untranslated region (UTR) region of *LATS2* and 5’tiRNA-His-GTG mimic. The sequence of the *ANG* promoter was cloned into vector pGL3-basic. The pGL3-ANG and Rluc plasmids were co-transfected into HCT116 cells, treated with a HIF-1α inhibitor and DMSO, respectively, and cultured in a hypoxia chamber. The 3’UTR sequence of *LATS2* was cloned into vector pmirGLO-basic. The negative control (NC) mimic or 5’tiRNA-His-GTG mimic were co-transfected with the wild-type or mutant vectors. After 48 h, the cells were harvested, and a luciferase assay was performed according to the manufacturer’s protocol (Promega, Madison, WI, USA). The relative value of luciferase was detected using a FLUOstar Omega (BMG LABTECH, Germany) and normalized to the value of Renilla luciferase activity.

### Immunofluorescence assay

Cells were grown on a chamber slide, cultured in normoxia or hypoxia chambers separately for 48 h, fixed with 4% paraformaldehyde, permeated with 0.3% Triton X-100, and blocked with 1% bovine serum albumin (BSA). The cells were then incubated with primary antibodies (ANG, 1:500, Abcam) and secondary antibodies (Alexa Fluor 488, 1:400, Thermo Fisher Scientific) according to the manufacturer’s protocol. Finally, the cell nuclei were counterstained using-(4-amidinophenyl)-1H-indole-6-carboxamidine (DAPI). The images were collected using a fluorescent microscope (Zeiss, Germany).

### Xenograft assay

All animal experiments were performed in strict accordance with institutional guidelines and approved by the Institutional Animal Care and Use Committee of the Shanghai Research Center for Model Organisms Inc.; the IACUC permit number was 2019–0023. An antagomir is an RNA oligonucleotide with specific modifications suitable for in vivo experiments, and we confirmed that the 5’tiRNA-His-GTG antagomir performs the same function as the 5’tiRNA-His-GTG inhibitor in HCT116 cells (Fig. S3). Then, HCT116 cells (2.5 × 10^6^ in 100 μL phosphate-buffered saline (PBS)) were injected subcutaneously through the right axilla of 5-week-old male BALB/c nude mice (6 mice per group), which were purchased from VRL Animal Technology (Beijing, China). Six days after tumor cell inoculation, mice were randomly divided into five groups, including PBS, NC antagomir (5 nmol each), Scramble antagomir (5 nmol each), 5’tiRNA-His-GTG agomir (5 nmol each), and 5’tiRNA-His-GTG antagomir (5 nmol each). The five groups of mice were treated with RNA-oligos by way of multiple-center intratumor injection four times every 3 days. The tumor size was measured using calipers every 3 days. After 3 weeks, the mice were sacrificed, and xenografts were then collected for weight measurements and other experiments. The schematic diagram of the animal experiments is shown in Fig. S4.

### Northern blotting

Northern blotting was performed to verify the results of qRT-PCR for 5’tiRNA-His-GTG and tRNA-His-GTG. The total RNA (20 μg) was separated by 15% urea acrylamide-polyacrylamide gels, then the separated gel was transferred onto the pre-wet Hybond-N+ nylon membrane (Millipore). The membrane was then UV-crosslinked at 1200 mJ in Stratagene UV Stratalinker 1800 (SCIENTZ03-II, Ningbo, China) and hybridized with DIG-labeled DNA probes (Sangon Biotech, Shanghai, China). The sequences of the northern probes are listed in Table S5.

### Statistical analysis

All results were obtained from at least three independent experiments and are expressed as means ± SD. All analyses were performed using GraphPad Prism 7.0 (GraphPad Software, USA). A two-tailed Student’s t-test was used to compare normally distributed continuous variables between two groups. The relationship between 5’tiRNA-His-GTG and tumor size was determined using Pearson’s correlation coefficient. *p* < 0.05 was considered statistically significant.

## Results

### 5’tiRNA-His-GTG is upregulated in human CRC tissues and is associated with tumor size

**Fig. 1:**
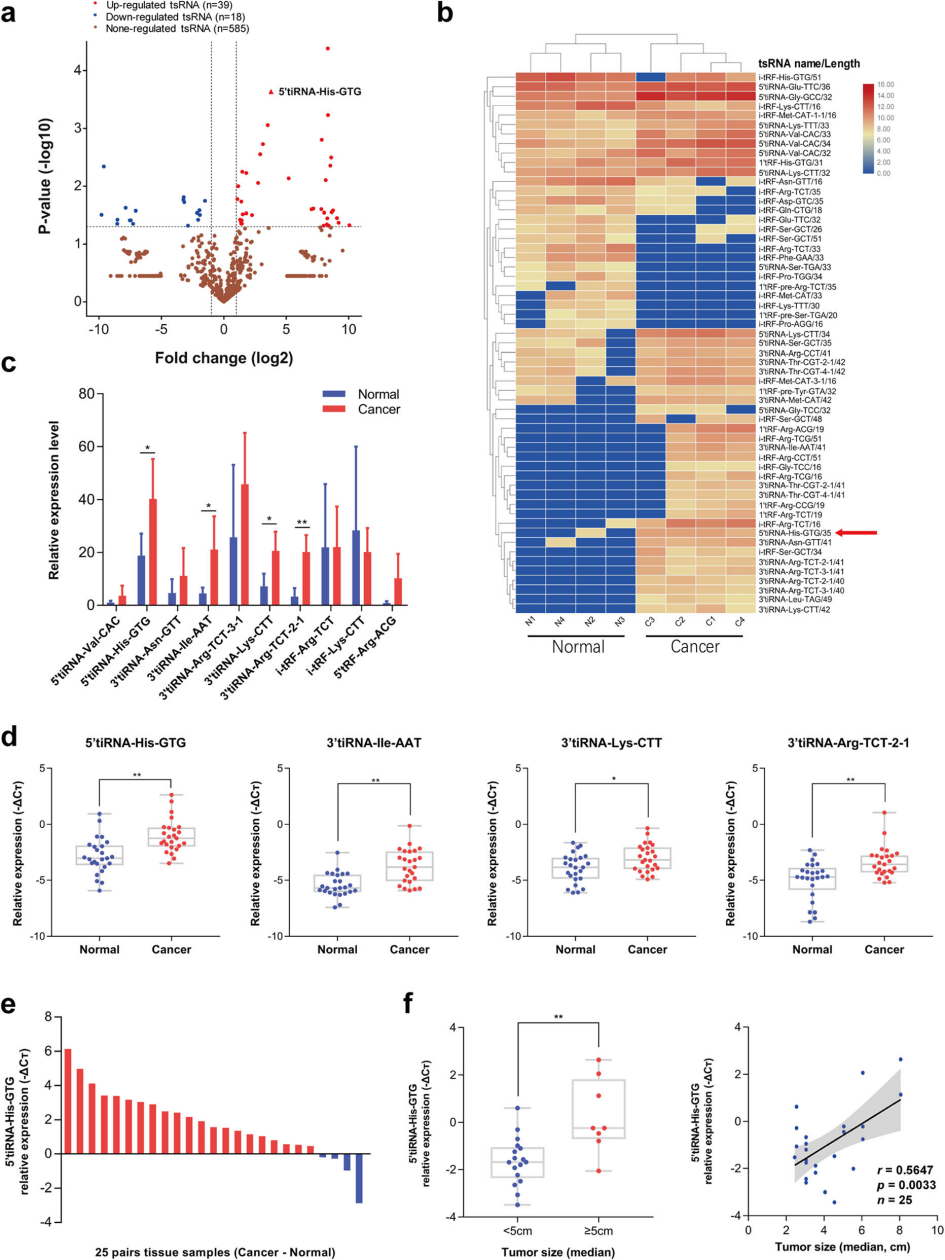
5’tiRNA-His-GTG is overexpressed in CRC tissues and is associated with tumor size. **a** Volcano plot displaying differentially expressed tsRNAs between four pairs of CRC and corresponding normal tissues. **b** Hierarchical cluster heatmap of upregulated and downregulated tsRNAs. **c** The relative expression levels of 10 selected tsRNAs were examined in four pairs of CRC and corresponding normal tissues using qRT-PCR. **d** The expression of 5’tiRNA-His-GTG, 3’tiRNA-Ile-AAT, 3’tiRNA-Lys-CTT, and 3’tiRNA-Arg-TCT-2-1 in 25 pairs of tissues were determined using qRT-PCR. **e** Paired comparison of 5’tiRNA-His-GTG expression levels between CRC and corresponding normal tissue (cancer-normal). **f** 5’tiRNA-His-GTG was highly expressed in larger tumors (left) and correlated positively with tumor size (right). *p < 0.05, **p < 0.01. All data are representative of at least three independent experiments and are presented as the means ± SD. CRC, colorectal cancer; tsRNA, tRNA-derived small RNA; qRT-PCR, quantitative real-time reverse transcription PCR

To identify essential tsRNAs expressed in CRC tissues, four pairs of CRC and corresponding normal tissues were selected for next-generation sequencing analysis. Dysregulation of tsRNAs were observed in CRC tissues (fold change ≥ 2 or < 0.5 and *p*-value < 0.05), with 39 upregulated tsRNAs and 18 downregulated tsRNAs (Fig. 1a, b). We then selected 10 of them for further qRT-PCR validation. The results showed that 5’tiRNA-His-GTG, 3’tiRNA-Ile-AAT, 3’tiRNA-Lys-CTT, and 3’tiRNA-Arg-TCT-2-1 were upregulated in CRC tissues (Fig. 1c). An additional 21 pairs of CRC and corresponding normal tissues were analyzed using qRT-PCR, which confirmed the above findings (Fig. 1d). Notably, the expression level of 5’tiRNA-His-GTG was the highest and upregulated by 3.5-fold in tumor tissues (Fig. 1e); besides, 5’tiRNAs have been reported to be more biologically significant compared with 3’tiRNAs [26, 27], and to play roles in tumor development [28]. Therefore, we focused on 5’tiRNA-His-GTG, which we considered to be an important tsRNA in the development of CRC, for further research. Moreover, we found that 5’tiRNA-His-GTG correlated positively with tumor size (Fig. 1f), but not with age, sex, or clinical stage (Fig. S1).

### Characteristics and detection methods of 5’tiRNA-His-GTG

**Fig. 2:**
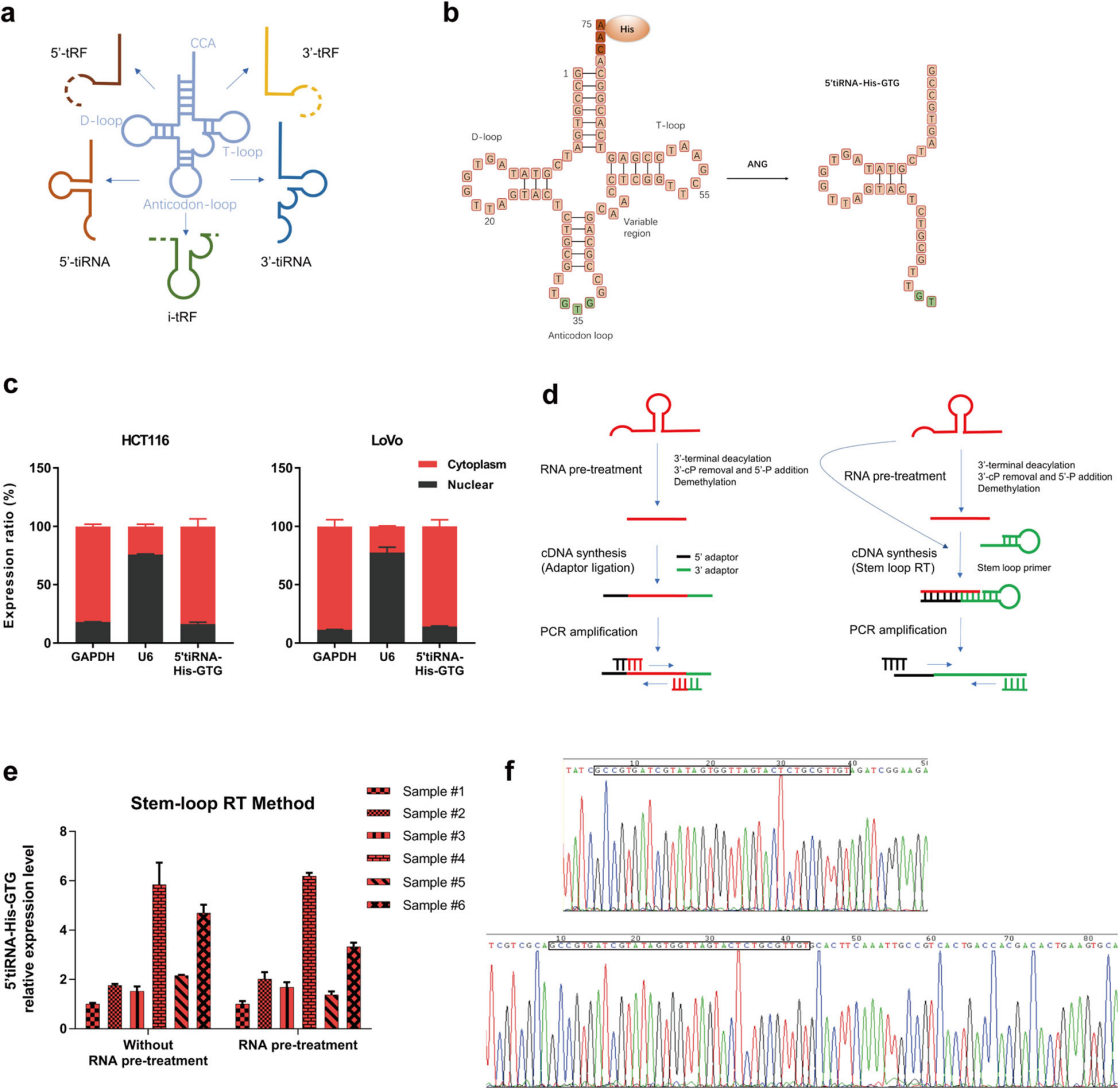
Characteristics and detection methods of 5’tiRNA-His-GTG. **a** tsRNAs are mainly divided into five types: i-tRF, 3′-tRF, 5′-tRF, 3′-tiRNA, and 5′-tiRNA. **b** 5’tiRNA-His-GTG was derived from mature tRNA-His-GTG with a length of 35 nt. **c** 5’tiRNA-His-GTG is mainly located in the cytoplasm, as assessed using a nuclear-cytoplasmic fractionation assay. **d** Schematic diagram of the two methods for detecting 5’tiRNA-His-GTG. **e** Relative expression level of 5’tiRNA-His-GTG with and without RNA pre-treatment using the Stem-loop RT method. **f** The product of qRT-PCR was confirmed by Sanger Sequencing. All data are representative of at least three independent experiments and are presented as the means ± SD. tsRNA, tRNA-derived small RNA; tRF, tRNA-derived fragment; tiRNA, tRNA-derived and stress-induced small RNA; qRT-PCR, quantitative real-time reverse transcription PCR

Currently, tsRNAs are classified into two major types: tiRNAs (5’tiRNAs and 3’tiRNAs) and tRFs (5’tRFs, 3’tRFs, and 1’tRFs), based on their enzyme cleavage sites (Fig. 2a). 5’tiRNA-His-GTG, derived from the half of mature tRNA-His-GTG, is a 5’tiRNA with a length of 35 nucleotides (Fig. 2b). To determine the intracellular location of 5’tiRNA-His-GTG, a nuclear-cytoplasmic fractionation assay was used to separate RNA from the cytoplasm and nucleus. qRT-PCR analysis showed that 5’tiRNA-His-GTG was mainly located in the cytoplasm in the indicated CRC cell lines (Fig. 2c).

tsRNAs are heavily decorated by RNA modifications that interfere with cDNA synthesis, and many tsRNAs share the same base sequence; therefore, the current detection method for tsRNAs requires additional de-modification and 3′/5′-adaptor ligation (Fig. 2d, left). However, 5’tiRNA-His-GTG only needed 3′-adaptor ligation, so we decided to adopt a simplified cDNA synthesis method called Stem-loop RT PCR (Fig. 2d, right). Moreover, we also found that RNA pre-treatment was not necessary to detect 5’tiRNA-His-GTG using the Stem-loop RT method (Fig. 2e). Finally, we performed the Sanger sequencing of the PCR products of qRT-PCR and Stem-loop RT PCR, and the sequences matched perfectly (Fig. 2f, Fig. S2). Northern blot also proved this simple method to detect 5’tiRNA-His-GTG is accurate (Fig. S11b, d).

### 5’tiRNA-His-GTG plays an oncogenic role in CRC

**Fig. 3:**
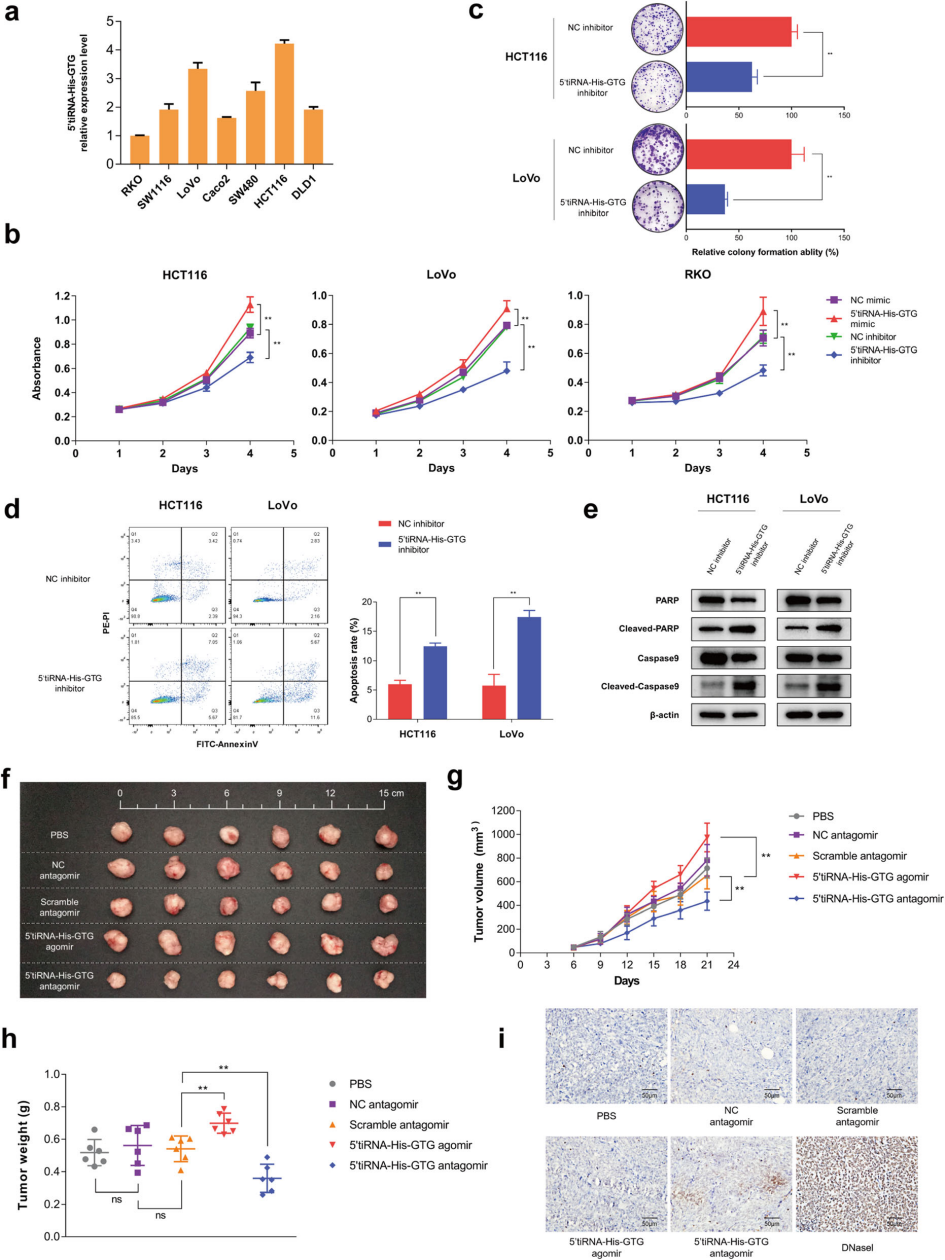
5’tiRNA-His-GTG plays an oncogenic role in CRC. **a** 5’tiRNA-His-GTG endogenous expression in CRC cell lines, as assessed using qRT-PCR. **b** The 5’tiRNA-His-GTG mimic promoted cell reproductive capacity, while the 5’tiRNA-His-GTG inhibitor significantly reduced cell proliferation of HCT116, LoVo, and RKO cells, as assessed using CCK8 assays. **c** 5’tiRNA-His-GTG inhibition reduced anchorage-dependent growth. **d** 5’tiRNA-His-GTG inhibition induced apoptosis, as assessed using FACS analysis. **e** Western blotting analysis showing the levels of apoptosis indicators (PARP, Cleaved-PARP, Caspase9, Cleaved-Caspase9) in 5’tiRNA-His-GTG inhibitor-treated HCT116 and LoVo cells. **f** At the end of the xenograft experiment, tumors from the five groups were dissected and photographed. **g** Tumor growth summarized using a line chart. **h** Tumor weights are shown in a histogram. **i** Representative TUNEL assay results. Apoptotic cells have brown-stained nuclei (the DNase I group is the positive control). *p < 0.05, **p < 0.01. All data are representative of at least three independent experiments and are presented as the means ± SD. CRC, colorectal cancer; qRT-PCR, quantitative real-time reverse transcription PCR; CCK8, cell counting kit 8; FACS, fluorescence activated cell sorting; PARP, poly (ADP-Ribose) polymerase 1; TUNEL, terminal deoxynulceotidyl transferase nick-end-labeling

The expression levels of 5’tiRNA-His-GTG in different CRC cell lines (RKO, SW1116, LoVo, Caco2, SW480, HCT116, and DLD1) were determined using qRT-PCR, and the results showed that 5’tiRNA-His-GTG is highly expressed in HCT116 and LoVo cells, and weakly in RKO cells (Fig. 3a). Inhibition and upregulation of 5’tiRNA-His-GTG in HCT116, LoVo, and RKO cells (the transfection efficiency is shown in Fig. S5a) were performed to explore the effect of 5’tiRNA-His-GTG on CRC cell proliferation. The results showed that transfection of 5’tiRNA-His-GTG mimic promoted cancer cell proliferation, while suppression of 5’tiRNA-His-GTG significantly inhibited cancer cell growth (Fig. 3b). Moreover, inhibition of 5’tiRNA-His-GTG markedly reduced colony formation (Fig. 3c, Fig. S6a) and induced apoptosis in the indicated cells (Fig. 3d, Fig. S6b). The induction of apoptosis was confirmed using western blotting. The levels of cleaved-poly (ADP-Ribose) polymerase 1 (PARP) and cleaved-Caspase 9 were upregulated after 5’tiRNA-His-GTG inhibition, while the levels of PARP and Caspase 9 were downregulated (Fig. 3e, Fig. S6c). Furthermore, to address the role of 5’tiRNA-His-GTG in vivo, 2.5 × 10^6^ HCT116 cells were injected into the right axilla subcutaneous tissues of nude mice. 6 days after inoculation, the tumors were treated with PBS, NC antagomir, Scramble antagomir, 5’tiRNA-His-GTG agomir, or 5’tiRNA-His-GTG antagomir via multiple-center intratumor injection five times every 3 days. Tumor growth was measured every 3 days and tumors were dissected at the end of the experiment. The size of the tumor in the 5’tiRNA-His-GTG agomir group was larger than that in other control groups, and the results of tumor growth and tumor weights showed that the 5’tiRNA-His-GTG agomir could increase the formation and growth of the transplanted tumors, while the 5’tiRNA-His-GTG antagomir had the opposite effect (Fig. 3f-h). Moreover, the 5’tiRNA-His-GTG antagomir could induce apoptosis, which was confirmed using a TUNEL assay (Fig. 3i). These results indicated that 5’tiRNA-His-GTG plays an oncogenic role in CRC progression.

### 5’tiRNA-His-GTG is regulated via the hypoxia/HIF1α/ANG axis

**Fig. 4:**
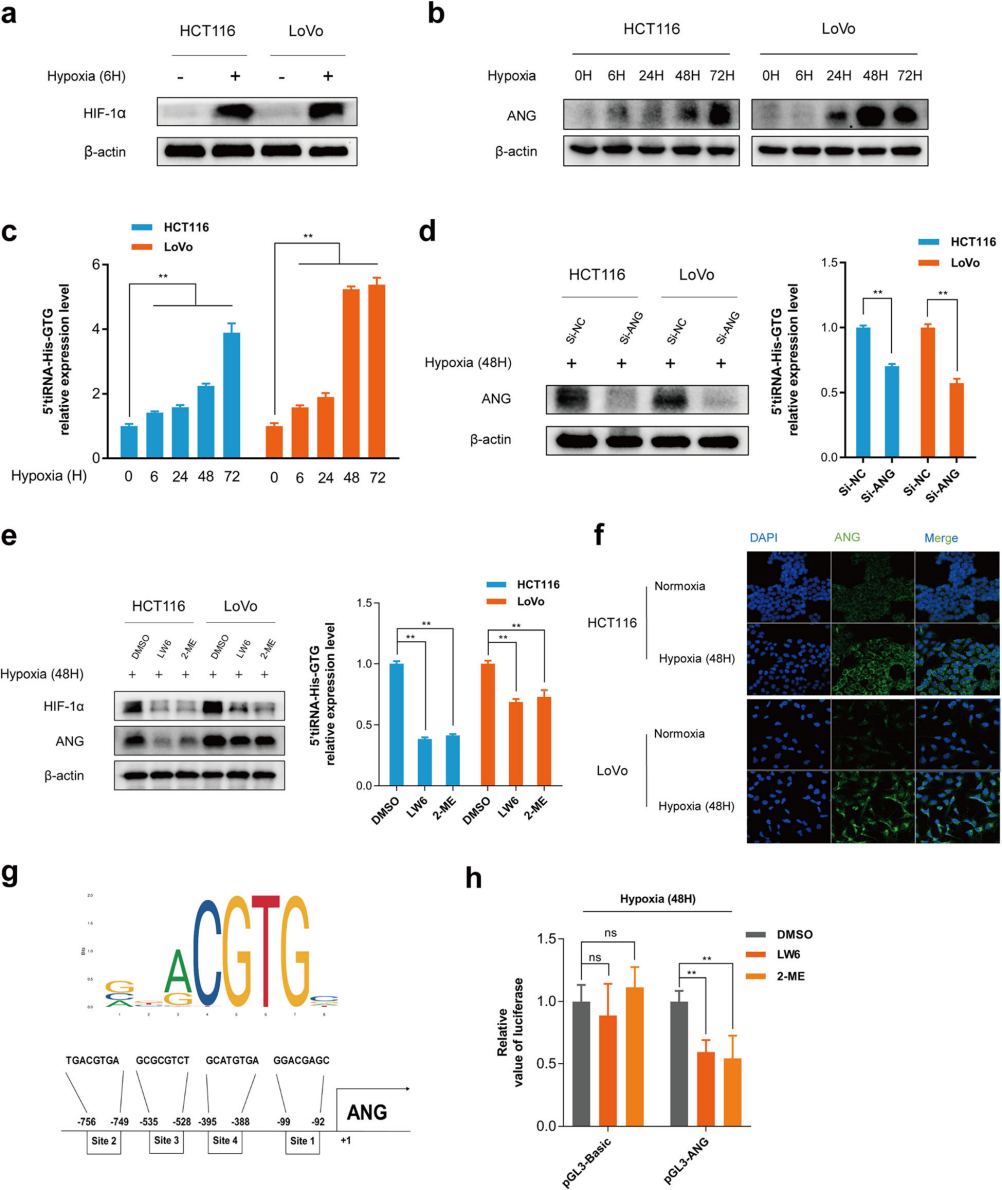
Hypoxia and HIF1α are involved in the regulation of 5’tiRNA-His-GTG expression. **a** HIF1α is overexpressed under hypoxic conditions, as determined using western blotting. **b** The level of ANG increased with prolonged hypoxia time. **c** The expression of 5’tiRNA-His-GTG is upregulated with prolonged hypoxia time, as determined using qRT-PCR. **d** ANG knockdown significantly reduced the level of the ANG protein (left) and the level of 5’tiRNA-His-GTG (right) **e** HIF1α inhibitors (LW6, 2-ME) significantly reduced the levels of HIF1α and ANG (left), as well as the 5’tiRNA-His-GTG expression level (right). **f** ANG is upregulated under hypoxic conditions and is mainly located in the cytoplasm of the indicated cells, as shown using immunofluorescence (IF). **g** The binding sites of HIF1α to the *ANG* promoter were obtained from JASPAR. **h** A luciferase reporter assay was used to measure the direct binding between HIF-1α and the *ANG* promoter region under a hypoxic environment. *p < 0.05, **p < 0.01, ns: not significant. All data are representative of at least three independent experiments and are presented as the means ± SD. ANG, angiogenin; HIF-1α, hypoxia inducible factor 1 subunit alpha; qRT-PCR, quantitative real-time reverse transcription PCR

The sustained rapid growth of cancer cells can outstrip the supply of nutrients and oxygen, creating a hypoxic environment [29], and a previous study showed that tiRNAs were induced under hypoxic conditions [30]. This might explain why 5’tiRNA-His-GTG is upregulated in tumor tissues and correlated positively with tumor size. To further explore this aspect, we cultured CRC cells in an anoxic incubator. The level of the HIF1α protein was significantly increased after 6 h of hypoxia in the indicated cells (Fig. 4a). Next, the expression levels of 5’tiRNA-His-GTG under hypoxic conditions were assessed and the result showed 5’tiRNA-His-GTG was upregulated with prolonged hypoxia time (Fig. 4c). ANG, which correlates closely with the production of tiRNAs [9], was determined in CRC cell lines under hypoxia. Notably, the protein (Fig. 4b) and mRNA levels (Fig. S5b) of ANG were increased under hypoxia. To further investigate the relationship between ANG and 5’tiRNA-His-GTG, an siRNA was used to knockdown *ANG* (the knockdown efficiency is shown in Fig. 4d and Fig. S5c) expression in cells cultured in the hypoxic environment. The expression level of 5’tiRNA-His-GTG decreased upon ANG depletion (Fig. 4d). Moreover, we found that HIF1α inhibitors (LW6 (10 μM) and 2-ME (10 μM)) suppressed both hypoxia-induced ANG and 5’tiRNA-His-GTG expression (Fig. S5d, Fig. 4e), which suggested that HIF1α was a critical upstream regulator of hypoxia-induced 5’tiRNA-His-GTG. Immunofluorescence analysis showed that ANG was upregulated under hypoxic conditions and is mainly located in the cytoplasm of the indicated cells (Fig. 4f), suggesting that the cleavage of tRNA-His-GTG into 5’tiRNA-His-GTG occurs in the cytosol. To further explore whether HIF1α affects ANG expression through transcriptional regulation, we identified four predicted binding sites for HIF1α in the *ANG* promoter (Fig. 4g). Luciferase assays revealed that the relative luciferase expression from the *ANG* promoter was reduced using HIF1α inhibitors under hypoxia (Fig. 4h). Finally, we proved that HIF1α inhibitors have no effect on HIF-1α/ANG/5’tiRNA-His-GTG axis under normoxia (Fig. S7). Taken together, the results suggested that 5’tiRNA-His-GTG is regulated via the hypoxia/HIF1α/ANG axis.

### 5’tiRNA-His-GTG directly targets LATS2 and inhibits hippo signaling pathway

**Fig. 5:**
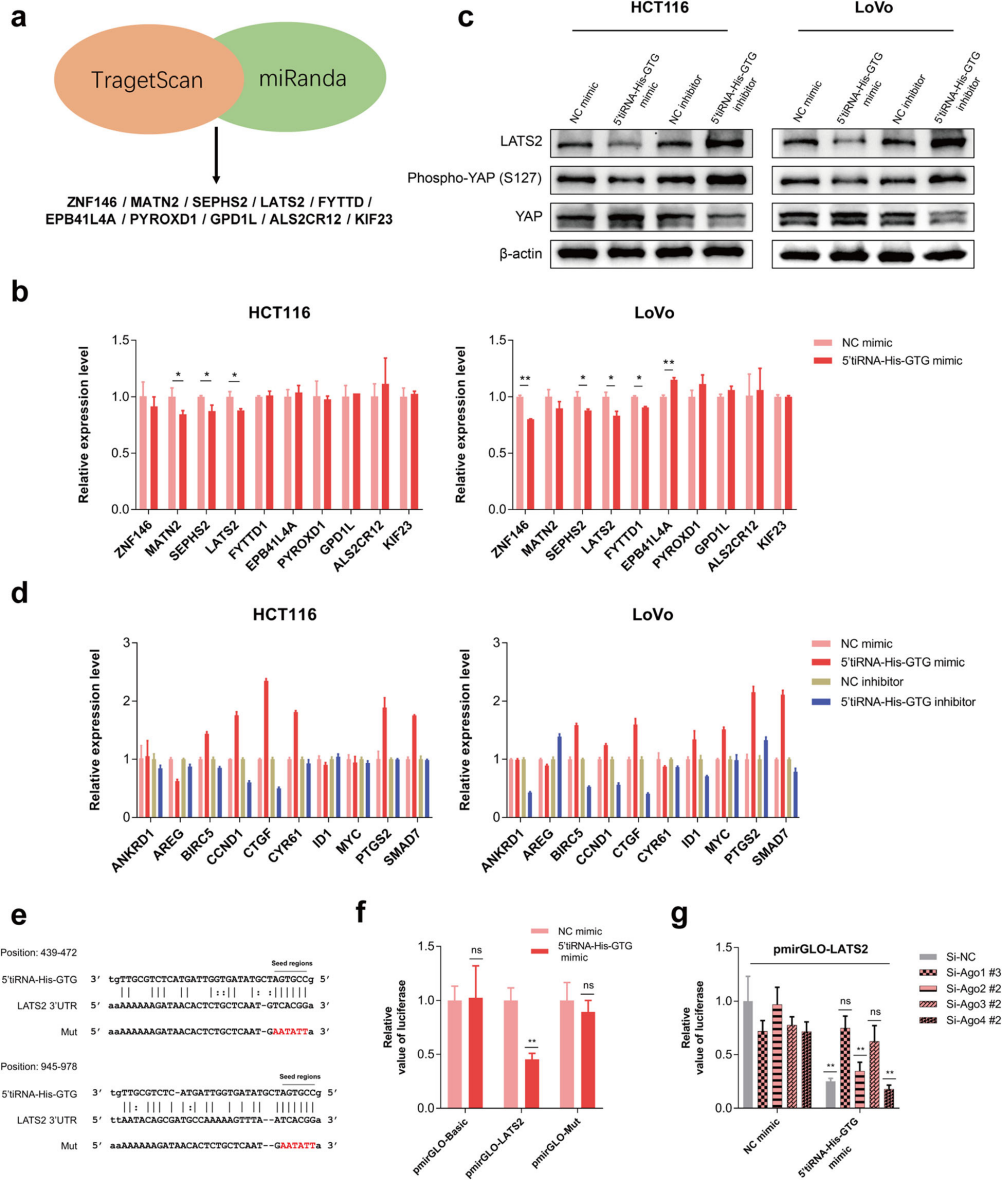
5’tiRNA-His-GTG is involved in the hippo signaling pathway by regulating LATS2 expression. **a** Schematic diagram of 5’tiRNA-His-GTG target prediction. **b** Experimental validation of the 10 highest-scoring 5’tiRNA-His-GTG target genes using qRT-PCR. **c** Western blot of LATS2, phospho-YAP (Ser127), and YAP expression upon 5’tiRNA-His-GTG mimic or inhibitor transfection in the indicated CRC cells. **d** Experimental validation of downstream genes of hippo signaling pathway using qRT-PCR. **e** Schematic diagram of the predicted interaction position between 5’tiRNA-His-GTG and the seed regions within the 3’UTR region and mutation region of *LATS2*. **f** The luciferase activity of pmirGLO-LATS2 was significantly decreased by the 5’tiRNA-His-GTG mimic in HCT116 cells. **g** HCT116 cells were co-transfected with a plasmid expressing the *LATS2* 3’UTR, 5’tiRNA-His-GTG mimic, and siRNAs targeting *AGO1*, *AGO2*, *AGO3*, and *AGO4*. After 48 h, transfected cells were collected with the luciferase assay. *p < 0.05, **p < 0.01, ns: not significant. All data are representative of at least three independent experiments and are presented as the means ± SD. LATS2, large tumor suppressor kinase 2; qRT-PCR, quantitative real-time reverse transcription PCR; YAP, Yes-associated protein; CRC, colorectal cancer; UTR, untranslated region; AGO, argonaute

We next investigated the molecular mechanism associated with 5’tiRNA-His-GTG in the progression of CRC. According to the principle of complementary base pairing, we used TargetScan and miRanda prediction tools to identify 10 potential target genes that are most likely to bind to 5’tiRNA-His-GTG (Table S6, Fig. 5a). To confirm the predicted target genes, 5’tiRNA-His-GTG mimics were transfected into the indicated cells, and after 48 h, the mRNA expression in the cells was analyzed using qRT-PCR. Among the 10 target genes, the expression levels of *LATS2* mRNA were decreased in both HCT116 and LoVo cells overexpressing 5’tiRNA-His-GTG (Fig. 5b). LATS2, a key component of the tumor-suppressive hippo signaling pathway, inhibits proliferation and promotes apoptosis by regulating yes-associated protein (YAP) activity [31, 32]. Therefore, the protein levels of LATS2, phosphorylated (phospho)-YAP (Ser127), and total YAP in the presence of 5’tiRNA-His-GTG mimics or inhibitors were detected. The results of western blotting revealed that the 5’tiRNA-His-GTG mimic significantly decreased the levels of LATS2 and phospho-YAP (Ser127), but increased the level of total YAP (Fig. 5c). In the xenograft mouse models, we also found that the level of LATS2 and phospho-YAP (Ser127) was decreased in the 5’tiRNA-His-GTG agomir group, while the level of total YAP was increased (Fig. S8). Considering that cell density might affect the expression of LATS2 [33], we examined the level of the LATS2 protein at different cell densities and found that high cell density increased the level of LATS2 (Fig. S9a). Therefore, the effect of 5’tiRNA-His-GTG mimic or inhibitor on the LATS2 protein is not caused by its effect on cell density. Besides, we found that hypoxic treatment decreased the expression of LATS2 (Fig. S9b) and reduced 5’tiRNA-His-GTG inhibition-mediated suppressive effects on CRC cells (Fig. S10a), and 5’tiRNA-His-GTG inhibitor attenuated hypoxia-induced YAP activation (Fig. S10b), indicating a close relationship between 5’tiRNA-His-GTG and LATS2 under hypoxia. To further demonstrate the impact of 5’tiRNA-His-GTG on the hippo signaling pathway, we validated 10 downstream genes of the hippo signaling pathway using qRT-PCR. The results indicated that 5’tiRNA-His-GTG affected many hippo signaling pathway-related genes such as *CTGF*, *BIRC5*, and *CCND1* (Fig. 5d). Target prediction programs indicated that there were potential specific targets for 5’tiRNA-His-GTG in the seed regions within the 3’UTR of *LATS2* (Fig. 5e). The empty vector, reporter vector pmirGLO carrying the *LATS2* 3’UTR, and the *LATS2* 3’UTR mutant vector were co-transfected with 5’tiRNA-His-GTG mimics or negative control into HCT116 cells. After 48 h, the cells were collected for luciferase detection. The results suggested that 5’tiRNA-His-GTG reduced the relative luciferase activity significantly when co-transfected with the pmirGLO-LATS2 compared with pmirGLO-basic or pmirGLO-mut (Fig. 5f). Previous studies have shown that argonaute (AGO) family proteins associate with tsRNAs and are involved in the regulation of gene expression [34, 35]. Therefore, we knocked down *AGO1*, *AGO2*, *AGO3*, and *AGO4* expression (Fig. S5e) and observed changes in luciferase expression. Luciferase expression inhibited by 5’tiRNA-His-GTG was reverted by the silencing of *AGO1* and *AGO3* (Fig. 5g). These results indicated AGO1 and AGO3 mediate 5’tiRNA-His-GTG regulation of *LATS2* gene expression.

### Attenuation of LATS2 partly rescues 5’tiRNA-His-GTG inhibition-mediated suppressive effects on CRC cells

**Fig. 6:**
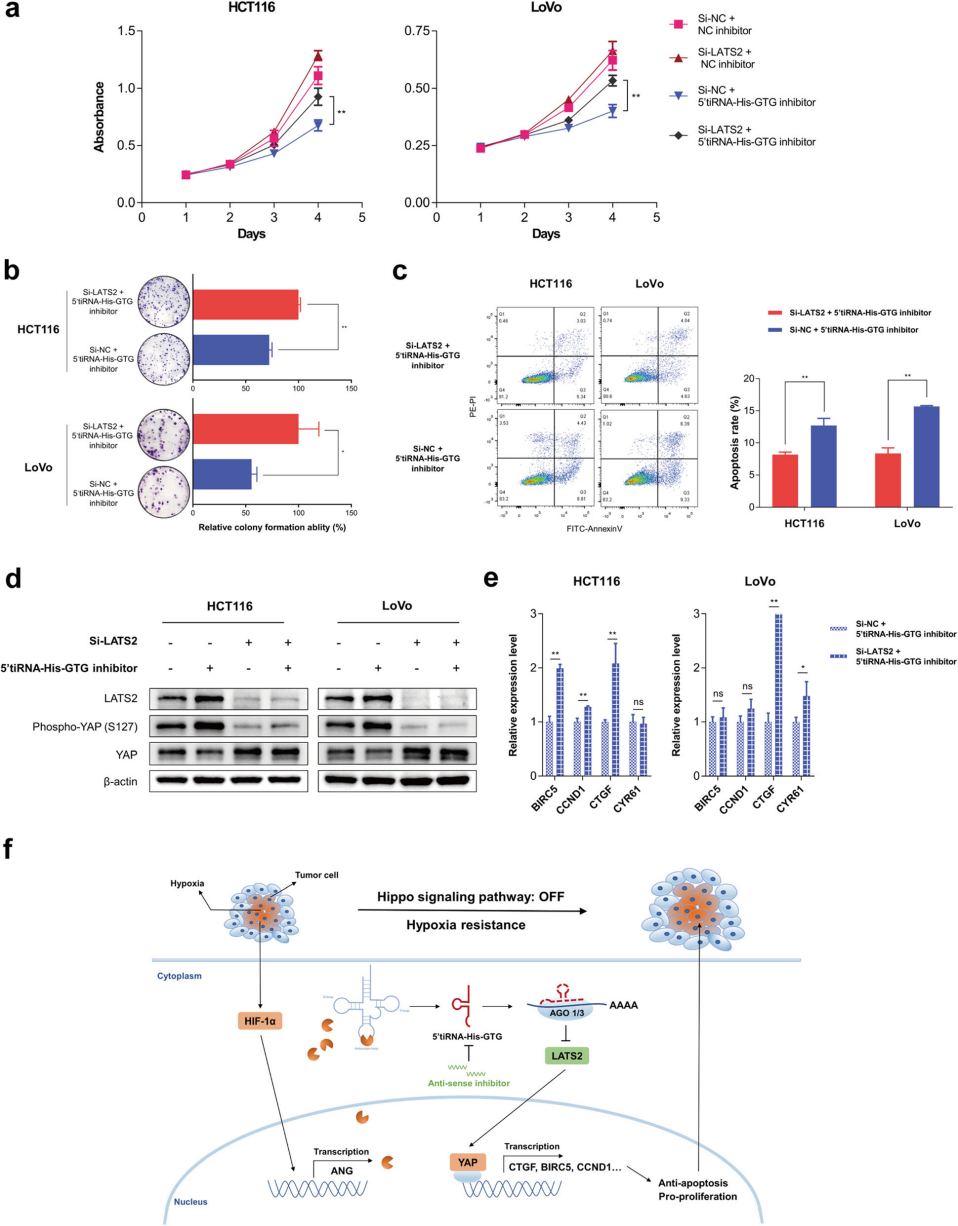
Attenuation of LATS2 partly rescues 5’tiRNA-His-GTG inhibition-mediated suppressive effects on CRC cells. **a** CCK8 assay showing inhibitor group increased cell proliferation in the Si-LATS2 / 5’tiRNA-His-GTG in HCT116 and LoVo cells, compared with that in the Si-NC / 5’tiRNA-His-GTG inhibitor group. **b** Colony formation assay showing that knockdown of LATS2 weakened the repressive effect of the 5’tiRNA-His-GTG inhibitor on colony formation. **c** FACS analysis showing that knockdown of *LATS2* reduced the cell apoptosis induced by the 5’tiRNA-His-GTG inhibitor. **d** Western blotting of LATS2, phospho-YAP (Ser127), and YAP expression upon Si-LATS2 or/and 5’tiRNA-His-GTG inhibitor transfection in the indicated CRC cells. **e** Some downstream genes of the hippo signaling pathway are upregulated, as measured using qRT-PCR. **f** Schematic diagram showing that hypoxia-induced 5’tiRNA-His-GTG mediates the expression of LATS2, thus contributing to the development of CRC. *p < 0.05, **p < 0.01, ns: not significant. All data are representative of at least three independent experiments and are presented as the means ± SD. LATS2, large tumor suppressor kinase 2; CRC, colorectal cancer; CCK8, cell counting kit 8; FACS, fluorescence activated cell sorting; YAP, Yes-associated protein

We next knocked down *LATS2* (the knockdown efficiency is shown in Fig. 6d and Fig. S5f) in 5’tiRNA-His-GTG-inhibited CRC cells and performed CCK8, colony formation, and apoptosis assays to investigate whether 5’tiRNA-His-GTG plays an oncogenic role in CRC progression dependent on LATS2. The CCK8 assay showed increased cell proliferation in the Si-LATS2 / 5’tiRNA-His-GTG inhibitor group in HCT116 and LoVo cells compared with the Si-NC / 5’tiRNA-His-GTG inhibitor group, but did not reach the level of the Si-NC / NC inhibitor group (Fig. 6a). Knockdown of *LATS2* could weaken the repressive effect of the 5’tiRNA-His-GTG inhibitor on colony formation (Fig. 6b), while it reduced the cell apoptosis induced by the 5’tiRNA-His-GTG inhibitor, as shown by FACS analysis (Fig. 6c). Knockdown of *LATS2* also decreased YAP phosphorylation, but increased the total level of YAP (Fig. 6d), as assessed using western blotting. Meanwhile, certain downstream genes of the hippo signaling pathway were upregulated upon Si-LATS2 transfection in 5’tiRNA-His-GTG-inhibited CRC cells (Fig. 6e). Taken together, these data demonstrated that LATS2 is involved in the suppressive effects of 5’tiRNA-His-GTG inhibition on CRC cells; however, the role of 5’tiRNA-His-GTG in CRC is not completely dependent on LATS2.

## Discussion

On the basis of our findings, we present a novel pathway (Fig. 6f) involved in the progression of CRC. In solid tumors, including CRC, sustaining rapid growth of cancer cells can outstrip the supply of nutrients and oxygen, creating a hypoxic environment. Hypoxia-induced HIF1α promotes the transcription of ANG, which in turn increases the cytoplasmic level of 5’tiRNA-His-GTG. Then, 5’tiRNA-His-GTG was found to “turn off” the hippo signaling pathway by inhibiting LATS2 translation. This discovery indicated that CRC cell might protect itself from the hypoxic environment by producing 5’tiRNA-His-GTG, which provides a new mechanism of CRC progression and updates strategies to control CRC.

tsRNAs, which were previously considered to be random degradation products of tRNAs, are now classified as an important class of functional small non-coding RNAs. However, our knowledge of tsRNAs is only the tip of the iceberg, there remain plentiful problems that need to be solved. For instance, the details of tsRNA generation are still largely unknown. Although previous studies showed that the production of tiRNAs is closely related to ANG [27, 36, 37], a recent study verified that the production of tiRNA is not completely dependent on ANG, suggesting there are other RNases that produce tiRNAs [38]. Moreover, mature tRNA has a lot of base modifications, such as m5C, m1A, m3C, and Ψ (Pseudouridylation), which can protect tRNAs from cleavage [39–43]. It is also essential to understand the intrinsic and extrinsic factors that participate in the production of tsRNAs. While our results stress that hypoxia/ HIF1α/ANG axis has a great impact on mature tRNA-His-GTG cleavage, which suggested that the cancer cells produced more 5’tiRNA-His-GTG under the external stimulation of hypoxia. Second, the current method for detecting tsRNAs is complex. Northern blot has been regarded as the gold standard for detecting tsRNA, but the process is complicated, and it is not applicable for screening from numerous tsRNAs. As for next-sequencing and qRT-PCR, tsRNAs need to erase their modifications first, such as m1A, m1G, m3C, which can interfere with reverse transcription [44]. Nonetheless, our data showed that the relative expression level of 5’tiRNA-His-GTG was consistent between the treated and non-treated groups. This may be due to insufficient methylation modification on 5’tiRNA-His-GTG, which also indicates that not every tsRNA needs to be demethylated before reverse transcription. Third, the relationship between base modifications on tsRNA and its biological function is currently unclear. However, existing evidence suggests that synthetic tsRNA is less functional than endogenous tsRNA [45]. It also needs to be mentioned that many of tsRNAs derived from 5′ end of tRNA (tRNA-Ala/Cys/Val) containing a stretch of guanine residues, which will form G-quadruplex and play a biological function such as inhibiting translation [42, 46, 47]. There is no evidence that 5’tiRNA-His-GTG can form G-quadruplex. Four, based on their source and length, tsRNAs have a wide variety and possess various biological functions. It seems that tiRNAs competitively bind to translation-related proteins, thereby inhibiting translation [26, 47], while tRFs were proved to regulate target gene expression based on sequence complementary like miRNA [18, 19, 48, 49]. More research is needed to reveal the inner nature of tsRNAs. Moreover, with the in-depth research on this kind of novel small non-coding RNAs, it is urgent to develop a systematic and unified naming method for tsRNAs.

In the present study, we sequenced four pairs of CRC and corresponding normal tissues to measure the global abundance of tsRNAs in CRC, which identified 39 upregulated tsRNAs and 18 downregulated tsRNAs. Intriguingly, 5’tiRNA-His-GTG was significantly upregulated by 3.5-fold in CRC tissues and correlated positively with tumor size. 5’tiRNA-His-GTG is a specific tRNA half derived from mature tRNA-His-GTG and mainly located in the cytoplasm. Cell experiments showed that 5’tiRNA-His-GTG is highly expressed in HCT116 and LoVo cell lines and is essential for cancer cell proliferation. Blocking 5’tiRNA-His-GTG can induce cell apoptosis, and we excluded the effect of 5’tiRNA-His-GTG inhibitor on mature tRNA-His-GTG via qRT-PCR (Fig. S11a, c) and northern blotting (Fig. S11d). Then, a xenograft experiment was performed to confirm the above findings, which showed that delivery of an antagomir against 5’tiRNA-His-GTG reduced the tumor burden and promoted apoptosis in mice xenografts, while the 5’tiRNA-His-GTG agomir could promote tumor growth. These findings indicated that 5’tiRNA-His-GTG acts as a tumor promoter in the progression of CRC. Next, the upstream regulatory mechanism of 5’tiRNA-His-GTG and its relationship with tumor size were studied. Previous studies showed that the production of tiRNAs is closely related to ANG [27, 37, 50, 51], and ANG is upregulated under hypoxic conditions [52, 53]. It is also reported that ANG is upregulated in CRC tissues [37]. Thus, we suspect that the upregulation of 5’tiRNA-His-GTG in CRC tissues was probably caused by an increase in ANG in response to the hypoxic environment in tumor tissues. 5’tiRNA-His-GTG is mainly located in the cytoplasm, providing an important clue to its biological origin and function. Our results suggested that ANG induced the production of 5’tiRNA-His-GTG under hypoxia. In addition, we found that HIF1α, the well-known sensor for cells to respond to oxygen availability [54, 55], was also involved in the production of ANG and 5’tiRNA-His-GTG. Luciferase reporter assays suggested that HIF1α might bind to the promoter region of *ANG* and promote its transcription. Overall, we showed that 5’tiRNA-His-GTG is regulated via the hypoxia/HIF1α/ANG axis.

We further explored the mechanisms by which 5’tiRNA-His-GTG contributes to colorectal carcinogenesis. Current research suggests that tsRNAs are involved in various cellular processes, including translational reprogramming [27, 36], apoptosis suppression [56, 57], and gene silencing [19, 49]. Additionally, some tsRNAs are bound by AGO proteins, which might employ a miRNA-like mechanism to regulate gene expression [34, 35, 49, 58]. Thus, using TargetScan and miRanda prediction tools, LATS2 was selected as the potential target of 5’tiRNA-His-GTG. LATS2, a pivotal kinase of the hippo signaling pathway, ensures the spatial and temporal control of yes-associated protein (YAP) activity, while YAP recruits other factors to induce gene transcription [59]. We found 5’tiRNA-His-GTG could significantly decrease the level of the LATS2 protein, but only slightly downregulated the expression of the *LATS2* mRNA. Moreover, the luciferase assay further revealed AGO1 and AGO3 mediate 5’tiRNA-His-GTG regulation of LATS2 expression. We hypothesized that 5’tiRNA-His-GTG could regulate the translation of *LATS2* rather than its transcription based on the following two points: 5’tiRNA-His-GTG has a special spatial structure (D-loop or some modifications) and cannot fully bind to the *LATS2* mRNA; in the AGO protein family, only AGO2 has strong enzymatic cleavage activity. Finally, rescue experiments showed that attenuation of LATS2 partly rescues 5’tiRNA-His-GTG inhibition-mediated suppressive effects on CRC cells. This confirmed the role of LATS2 in 5’tiRNA-His-GTG-mediated carcinogenesis.

Our study also has several limitations. First, although we found that four tiRNAs were upregulated in CRC tissues, only 5’tiRNA-His-GTG was uncovered. The role of 3’tiRNA-Ile-AAT, 3’tiRNA-Lys-CTT, and 3’tiRNA-Arg-TCT-2-1 need to be further studied. Second, ANG is well-known for promoting tumor angiogenesis under hypoxia [60], but it is reported that ANG-induced tsRNAs (derived from tRNA-Val-CAC and tRNA-Gly-GCC) act as negative regulators of angiogenesis in endothelial cells under hypoxia [61]. Considering that ANG can induce the generation of various tsRNAs, whether these various tsRNAs, including 5’tiRNA-His-GTG, play different roles in tumor angiogenesis requires further investigations. Third, 5’tiRNA-His-GTG is larger than miRNA, whether it can directly bind to AGO1/3 and be loaded into the RNA-induced silencing complex (RISC) need more explorations. Fourth, attenuation of LATS2 could not completely rescue 5’tiRNA-His-GTG inhibition-mediated suppressive effects on CRC cells, thus there remain other targets of 5’tiRNA-His-GTG, which may be related to the inhibition of translation initiation by 5’tiRNA [26]. Our research provides the basis for further in-depth studies of 5’tiRNA-His-GTG in CRC.

## Conclusions

In conclusion, we identified 5’tiRNA-His-GTG as an oncogenic tRNA half in CRC and further uncovered a novel pathway in which hypoxia-induced 5’tiRNA-His-GTG suppresses LATS2, leading to CRC progression. Targeting 5’tiRNA-His-GTG or its associated network could represent novel therapies for patients with CRC.

## Supplementary Information

**Additional file 1: Table S1**. Clinical information for the patients included in this study. **Table S2**. Primers used for real-time PCR assay. **Table S3**. Small interfering RNA (siRNA) sequences. **Table S4**. RNA oligonucleotide sequences. **Table S5**. Northern blot probe sequences. **Table S6**. Predicted target genes of 5’tiRNA-His-GTG. **Figure S1**. Distribution of the 5’tiRNA-His-GTG relative expression level in different groups. (a) The expression level of 5’tiRNA-His-GTG showed no significant difference between early stage and advanced stage CRC. (b) There was no difference in the expression level of 5’tiRNA-His-GTG above and below the age of 60 years old. (c) The expression level of 5’tiRNA-His-GTG was not significantly different between males and females. ns: not significant. CRC, colorectal cancer. **Figure S2**. Sanger sequencing of the PCR products of qRT-PCR. (a) 3’tiRNA-Lys-CTT. (b) 3’tiRNA-Arg-TCT-2-1. (c) 3’tiRNA-Ile-AAT. **Figure S3**. The 5’tiRNA-His-GTG antagomir performs the same function as 5’tiRNA-His-GTG inhibitor in HCT116 cells. **Figure S4**. Schematic diagram of animal experiments. **Figure S5**. qRT-PCR validation of different genes. **Figure S6**. 5’tiRNA-His-GTG inhibition reduces anchorage-dependent growth and induces apoptosis in RKO cells. **Figure S7**. LW6 and 2-ME have no effect on HIF-1α/ANG/5’tiRNA-His-GTG axis under normoxia in HCT116 cells. **Figure S8**. The expression level of LATS2, phospho-YAP (Ser127), and YAP in the xenograft tumors. **Figure S9**. The expression level of LATS2 upon different cell densities and various hypoxia time. **Figure S10**. The role of 5’tiRNA-His-GTG inhibitor under hypoxic environment. **Figure S11**. The expression level of tRNA-His-GTG and 5’tiRNA-His-GTG in various groups using qRT-PCR and northern blot.

## Data Availability

The datasets used and/or analyzed during the current study are available from the corresponding author on reasonable request.

## Abbreviations

AGO: Argonaute
ANG: Angiogenin
CRC: Colorectal cancer
HIF1α: Hypoxia inducible factor 1 subunit alpha
LATS2: Large tumor suppressor kinase 2
ncRNAs: Non-coding RNAs
PARP: Cleaved poly (ADP-Ribose) polymerase 1
RISC: RNA-induced silencing complex
tiRNAs: tRNA-derived and stress-induced small RNAs
tRFs: tRNA fragments
tsRNAs: tRNA-derived small RNAs
YAP: Yes-associated protein
YBX1: Y box binding protein 1
