# Supplementary material for: A specific tRNA half, 5’tiRNA-His-GTG, responds to hypoxia via the HIF1α/ANG axis and promotes colorectal cancer progression by regulating LATS2
Source: J Exp Clin Cancer Res. 2021 Feb 15;40:67. doi: 10.1186/s13046-021-01836-7 (PMC7885485; doi:10.1186/s13046-021-01836-7)
Supplement: Supplementary file 1 — Additional file 1: Table S1. Clinical information for the patients included in this study. Table S2. Primers used for real-time PCR assay. Table S3. Small interfering RNA (siRNA) sequences. Table S4. RNA oligonucleotide sequences. Table S5. Northern blot probe sequences. Table S6. Predicted target genes of 5’tiRNA-His-GTG. Figure S1. Distribution of the 5’tiRNA-His-GTG relative expression level in different groups. (a) The expression level of 5’tiRNA-His-GTG showed no significant difference between early stage and advanced stage CRC. (b) There was no difference in the expression level of 5’tiRNA-His-GTG above and below the age of 60 years old. (c) The expression level of 5’tiRNA-His-GTG was not significantly different between males and females. ns: not significant. CRC, colorectal cancer. Figure S2. Sanger sequencing of the PCR products of qRT-PCR. (a) 3’tiRNA-Lys-CTT. (b) 3’tiRNA-Arg-TCT-2-1. (c) 3’tiRNA-Ile-AAT. Figure S3. The 5’tiRNA-His-GTG antagomir performs the same function as 5’tiRNA-His-GTG inhibitor in HCT116 cells. Figure S4. Schematic diagram of animal experiments. Figure S5. qRT-PCR validation of different genes. Figure S6. 5’tiRNA-His-GTG inhibition reduces anchorage-dependent growth and induces apoptosis in RKO cells. Figure S7. LW6 and 2-ME have no effect on HIF-1α/ANG/5’tiRNA-His-GTG axis under normoxia in HCT116 cells. Figure S8. The expression level of LATS2, phospho-YAP (Ser127), and YAP in the xenograft tumors. Figure S9. The expression level of LATS2 upon different cell densities and various hypoxia time. Figure S10. The role of 5’tiRNA-His-GTG inhibitor under hypoxic environment. Figure S11. The expression level of tRNA-His-GTG and 5’tiRNA-His-GTG in various groups using qRT-PCR and northern blot. [file 13046_2021_1836_MOESM1_ESM.docx]

**Supplemental Information**

**A specific tRNA half, 5’tiRNA-His-GTG, responds to hypoxia via the HIF1α/ANG axis and promotes colorectal cancer progression by regulating LATS2**

En-Wei Tao et, al.

**Supplemental tables**

**Table S1. Clinical information for the patients included in this study.**

| Patients | Gender | Age (years) | Tumor size (median, cm) | AJCC stage | Metastasis | tsRNA sequencing |
| --- | --- | --- | --- | --- | --- | --- |
| 1 | Male | 50 | 3 | Ⅲ | Yes | √ |
| 2 | Male | 59 | 3 | Ⅲ | Yes | √ |
| 3 | Male | 66 | 4 | Ⅳ | Yes | √ |
| 4 | Male | 71 | 3 | Ⅳ | Yes | √ |
| 5 | Male | 82 | 8 | Ⅲ | Yes | - |
| 6 | Male | 80 | 3 | Ⅱ | No | - |
| 7 | Female | 72 | 3.5 | Ⅱ | No | - |
| 8 | Male | 56 | 3 | Ⅲ | Yes | - |
| 9 | Female | 69 | 3 | Ⅱ | No | - |
| 10 | Male | 59 | 4.5 | Ⅱ | No | - |
| 11 | Male | 44 | 4.5 | Ⅱ | No | - |
| 12 | Female | 77 | 8 | Ⅳ | Yes | - |
| 13 | Female | 78 | 2.5 | Ⅱ | No | - |
| 14 | Female | 73 | 5 | Ⅱ | No | - |
| 15 | Female | 80 | 5.5 | Ⅱ | No | - |
| 16 | Male | 73 | 2.5 | Ⅱ | No | - |
| 17 | Male | 76 | 3 | Ⅲ | Yes | - |
| 18 | Male | 65 | 3.5 | Ⅱ | No | - |
| 19 | Male | 59 | 6 | Ⅱ | No | - |
| 20 | Male | 84 | 6 | Ⅲ | Yes | - |
| 21 | Male | 39 | 2.4 | Ⅱ | No | - |
| 22 | Male | 64 | 2.5 | Ⅱ | No | - |
| 23 | Male | 77 | 5 | Ⅱ | No | - |
| 24 | Male | 70 | 6 | Ⅱ | No | - |
| 25 | Female | 64 | 3 | Ⅳ | Yes | - |

**Table S2. Primers used for real-time PCR assay.**

| Gene | Forward (5'-3') | Reverse (5'-3') |
| --- | --- | --- |
| U6 | GCTTCGGCAGCACATATACTAAAAT | CGCTTCACGAATTTGCGTGTCAT |
| 5'tiRNA-Val-CAC | CGACGATCGCTTCTGTAGTGTAGT | GCTCTTCCGATCTGAGGCGA |
| 5'tiRNA-His-GTG | ATCGCCGTGATCGTATAGTGG | CTTCCGATCTACAACGCAGAGTAC |
| 3'tiRNA-Asn-GTT | ATCTAACCGAAAGGTTGGTGGT | TCTTCCGATCTTGGCGTCC |
| 3'tiRNA-Ile-AAT | CGACGATCTAACGCCAAGGT | TGTGCTCTTCCGATCTTGGTG |
| 3'tiRNA-Arg-TCT--3-1 | ATTCAAAGGTTGTGGGTTCGAA | CTCTTCCGATCTTGGCGACTC |
| i-tRF-Lys-CTT | GAGTTCTACAGTCCGACGATCCG | TTCCGATCTGTCCCATGCTCTA |
| 3'tiRNA-Arg-TCT--2-1 | ATTCAAAGGTTGTGGGTTCGAG | CTCTTCCGATCTTGGCGACTC |
| i-tRF-Arg-TCT | CTACAGTCCGACGATCTCAAAGG | TGTGCTCTTCCGATCTGACTCC |
| 3'tiRNA-Lys-CTT | CGATCTTAATCTCAGGGTCGTG | GATCTTGGCGCCCAACGT |
| 5'tRF-Arg-ACG | TACAGTCCGACGATCGGGCCAG | GCTCTTCCGATCTTCCATTGCG |
| β-actin | CACCATTGGCAATGAGCGGTTC | AGGTCTTTGCGGATGTCCACGT |
| GAPDH | GCATTGCCCTCAACGACCAC | CCACCACCCTGTTGCTGTAG |
| ANG | AGAAGCGGGTGAGAAACAAAAC | AGTGCTGGGTCAGGAAGTGTG |
| ZNF146 | GGAAAACCTCCTTACGCACCAG | CTCCTGTGTGAGTTCTCTGGTG |
| MATN2 | CTGAACACGGAGGATTCCTTCG | GGACACTGACAGGCAAAGGATC |
| SEPHS2 | TAGCTTGTGCCAACGTGCTGAG | TGAGTGGCGTTACCTTTTCGCG |
| LATS2 | GTTCTTCATGGAGCAGCACGTG | CTGGTAGAGGATCTTCCGCATC |
| FYTTD1 | CCAGCAATTCAGGATGAGAGTGC | CCAGTCGTTTTCCTAGCTGCAAG |
| EPB41L4A | CGAAGTTACCGCCAGTATCGCA | CAGAACCCTGAGCATCCGAAGA |
| PYROXD1 | GTCCTTCACTTTTCCAAGAGACC | GGTGTAACTCCTGTAGCACTGAC |
| GPD1L | CCGTGGTTGATGATGCAGACAC | CGCTTTGGTGTTGTCTCCACAG |
| ALS2CR12 | AAGTTGGCTGCCCAAGAGAAGC | CCATTTCGCCTTCTGTTTTGACC |
| KIF23 | GTAGCAAGACCTGTAGACAAGGC | TTCGCATGACGGCAAAGGTGGA |
| ANKRD1 | AGTAGAGGAACTGGTCACTGG | TGGGCTAGAAGTGTCTTCAGAT |
| AREG | GCACCTGGAAGCAGTAACATGC | GGCAGCTATGGCTGCTAATGCA |
| BIRC5 | CCACTGAGAACGAGCCAGACTT | GTATTACAGGCGTAAGCCACCG |
| CCND1 | TCTACACCGACAACTCCATCCG | TCTGGCATTTTGGAGAGGAAGTG |
| CTGF | AGGAGTGGGTGTGTGACGA | CCAGGCAGTTGGCTCTAATC |
| CYR61 | GGAAAAGGCAGCTCACTGAAGC | GGAGATACCAGTTCCACAGGTC |
| ID1 | GTTGGAGCTGAACTCGGAATCC | ACACAAGATGCGATCGTCCGCA |
| Myc | CCTGGTGCTCCATGAGGAGAC | CAGACTCTGACCTTTTGCCAGG |
| PTGS2 | CGGTGAAACTCTGGCTAGACAG | GCAAACCGTAGATGCTCAGGGA |
| SMAD7 | TGTCCAGATGCTGTGCCTTCCT | CTCGTCTTCTCCTCCCAGTATG |
| AGO1 | ACAGTGTCGAGAAGAGGTGCTC | GAGTAGGTGTTCTTGAGATGCCG |
| AGO2 | CAAGTCGGACAGGAGCAGAAAC | GACCTAGCAGTCGCTCTGATCA |
| AGO3 | CTTCTGTGTTCCAGCAACCAGTG | TGTGGCACAGTATCTGCTTGGG |
| AGO4 | ACAAGGTGCAGACAGTGTGGAG | TCTCCAACACGTTTCACCTCCG |

**Table S3. Small interfering RNA (siRNA) sequences.**

| Gene | Sense (5'-3') | Antisense (5'-3') |
| --- | --- | --- |
| NC siRNA | UUCUCCGAACGUGUCACGUTT | ACGUGACACGUUCGGAGAATT |
| ANG siRNA | AAACCUAAGAAUAAGCAAGUCAU | ATGACTTGCTTATTCTTAGGTTT |
| LATS2 siRNA | CCGCAAAGGGUACACUCAATT | UUGAGUGUACCCUUUGCGGAG |
| AGO1 siRNA#1 | GCCUCAGAUCUUUGGUGAUTT | AUCACCAAAGAUCUGAGGCTT |
| AGO1 siRNA#2 | GCUGGACAUCAGGAACAUATT | UAUGUUCCUGAUGUCCAGCTT |
| AGO1 siRNA#3 | GCUACAACUUAGAUCCCUATT | UAGGGAUCUAAGUUGUAGCTT |
| AGO2 siRNA#1 | GCGUUACACGAUGCACUUUTT | AAAGUGCAUCGUGUAACGCTT |
| AGO2 siRNA#2 | GCAAGAAGAGAUUAGCAAATT | UUUGCUAAUCUCUUCUUGCTT |
| AGO2 siRNA#3 | GGUACCACCUGGUGGAUAATT | UUAUCCACCAGGUGGUACCTT |
| AGO3 siRNA#1 | CCAUUAAACUGCUGGCUAATT | UUAGCCAGCAGUUUAAUGGTT |
| AGO3 siRNA#2 | GGUUCAAGCCUACUCGUAUTT | AUACGAGUAGGCUUGAACCTT |
| AGO3 siRNA#3 | GCUCACCUGGUAGCAUUUATT | UAAAUGCUACCAGGUGAGCTT |
| AGO4 siRNA#1 | GCACUUGAUGUUAUCACAATT | UUGUGAUAACAUCAAGUGCTT |
| AGO4 siRNA#2 | GGAUAGUACCAUCACACAUTT | AUGUGUGAUGGUACUAUCCTT |
| AGO4 siRNA#3 | GCACUCGCUCAGUCUCUAUTT | AUAGAGACUGAGCGAGUGCTT |

**Table S4. RNA oligonucleotide sequences.**

| Gene | Sequence (5'-3') | Modification |
| --- | --- | --- |
| 5'tiRNA-His-GTG mimic | GCCGUGAUCGUAUAGUGGUUAGUACUCUGCGUUGU | 2'Ome |
| 5'tiRNA-His-GTG inhibitor | ACAACGCAGAGUACUAACCACUAUACGAUCACGGC | 2'Ome |
| NC mimic | UUGUACUACACAAAAGUACUG | 2'Ome |
| NC inhibitor | CAGUACUUUUGUGUAGUACAA | 2'Ome |
| 5'tiRNA-His-GTG antagomir | ACAACGCAGAGUACUAACCACUAUACGAUCACGGC | Specific |
| 5'tiRNA-His-GTG agomir | GCCGUGAUCGUAUAGUGGUUAGUACUCUGCGUUGU | Specific |
| NC antagomir | CAGUACUUUUGUGUAGUACAAA | Specific |
| Scramble antagomir | GUUUUAGCAUGCGACUCAGAGCUGUCGUUGGUUGU | Specific |

**Table S5. Northern blot probe sequences.**

| Gene | Sequence (5'-3') |
| --- | --- |
| U6 | GTGCTCGCTTCGGCAGCACATATACTAAAATTGGAACGATACAGA GAAGATTAGCATGGCCCCTGCGCAAGGATGACACGCAAATTCGTG AAGCGTTCCATATTTT |
| 5'tiRNA-His-GTG | ACAACGCAGAGTACTAACCACTATACGATCACGGC |

**Table S6. Predicted target genes of 5’tiRNA-His-GTG**

| Target | | | TargetScan | | miRanda | |
| --- | --- | --- | --- | --- | --- | --- |
| Seqname | GeneSymbol | Type | Total | Context+ | Context | Structure |
| [NM_007145](http://www.ncbi.nlm.nih.gov/nuccore/NM_007145?report=genbank) | ZNF146 | Coding | 1 | -0.589 | -0.52 | 158 |
| [NM_002380](http://www.ncbi.nlm.nih.gov/nuccore/NM_002380?report=genbank) | MATN2 | Coding | 1 | -0.587 | -0.45 | 167 |
| [NM_012248](http://www.ncbi.nlm.nih.gov/nuccore/NM_012248?report=genbank) | SEPHS2 | Coding | 1 | -0.559 | -0.396 | 167 |
| [NM_014572](http://www.ncbi.nlm.nih.gov/nuccore/NM_014572?report=genbank) | LATS2 | Coding | 2 | -0.544 | -0.396 | 312 |
| [NM_032288](http://www.ncbi.nlm.nih.gov/nuccore/NM_032288?report=genbank) | FYTTD1 | Coding | 1 | -0.525 | -0.418 | 155 |
| [NM_022140](http://www.ncbi.nlm.nih.gov/nuccore/NM_022140?report=genbank) | EPB41L4A | Coding | 1 | -0.514 | -0.405 | 160 |
| [NM_024854](http://www.ncbi.nlm.nih.gov/nuccore/NM_024854?report=genbank) | PYROXD1 | Coding | 1 | -0.513 | -0.464 | 154 |
| [NM_015141](http://www.ncbi.nlm.nih.gov/nuccore/NM_015141?report=genbank) | GPD1L | Coding | 1 | -0.509 | -0.402 | 171 |
| [NM_001127391](http://www.ncbi.nlm.nih.gov/nuccore/NM_001127391?report=genbank) | ALS2CR12 | Coding | 1 | -0.507 | -0.386 | 149 |
| [NM_138555](http://www.ncbi.nlm.nih.gov/nuccore/NM_138555?report=genbank) | KIF23 | Coding | 1 | -0.503 | -0.381 | 156 |
| [NM_001288706](http://www.ncbi.nlm.nih.gov/nuccore/NM_001288706?report=genbank) | IL1R1 | Coding | 1 | -0.497 | -0.358 | 163 |
| [NM_000151](http://www.ncbi.nlm.nih.gov/nuccore/NM_000151?report=genbank) | G6PC | Coding | 1 | -0.495 | -0.386 | 143 |
| [NM_032936](http://www.ncbi.nlm.nih.gov/nuccore/NM_032936?report=genbank) | TMEM60 | Coding | 1 | -0.492 | -0.387 | 149 |
| [NM_181672](http://www.ncbi.nlm.nih.gov/nuccore/NM_181672?report=genbank) | OGT | Coding | 1 | -0.484 | -0.424 | 173 |
| [NM_030790](http://www.ncbi.nlm.nih.gov/nuccore/NM_030790?report=genbank) | ITFG1 | Coding | 1 | -0.475 | -0.403 | 160 |
| [NM_007108](http://www.ncbi.nlm.nih.gov/nuccore/NM_007108?report=genbank) | TCEB2 | Coding | 1 | -0.47 | -0.287 | 156 |
| [NM_058246](http://www.ncbi.nlm.nih.gov/nuccore/NM_058246?report=genbank) | DNAJB6 | Coding | 1 | -0.466 | -0.244 | 172 |
| [NM_001010863](http://www.ncbi.nlm.nih.gov/nuccore/NM_001010863?report=genbank) | C10orf128 | Coding | 1 | -0.46 | -0.348 | 153 |
| ENST00000399413 | AC129492.6 | NonCoding | 1 | -0.457 | -0.328 | 158 |
| [NM_014309](http://www.ncbi.nlm.nih.gov/nuccore/NM_014309?report=genbank) | RBFOX2 | Coding | 1 | -0.457 | -0.353 | 166 |
| [NM_020654](http://www.ncbi.nlm.nih.gov/nuccore/NM_020654?report=genbank) | SENP7 | Coding | 1 | -0.453 | -0.41 | 171 |
| [NM_006468](http://www.ncbi.nlm.nih.gov/nuccore/NM_006468?report=genbank) | POLR3C | Coding | 1 | -0.451 | -0.373 | 161 |
| [NM_145231](http://www.ncbi.nlm.nih.gov/nuccore/NM_145231?report=genbank) | EFCAB11 | Coding | 1 | -0.451 | -0.357 | 169 |
| [NM_001127](http://www.ncbi.nlm.nih.gov/nuccore/NM_001127?report=genbank) | AP1B1 | Coding | 1 | -0.45 | -0.339 | 157 |
| [NM_198321](http://www.ncbi.nlm.nih.gov/nuccore/NM_198321?report=genbank) | GALNT10 | Coding | 1 | -0.43 | -0.298 | 175 |
| [NM_004296](http://www.ncbi.nlm.nih.gov/nuccore/NM_004296?report=genbank) | RGS6 | Coding | 1 | -0.429 | -0.257 | 170 |
| [NM_014743](http://www.ncbi.nlm.nih.gov/nuccore/NM_014743?report=genbank) | KIAA0232 | Coding | 1 | -0.427 | -0.345 | 163 |
| [NM_015719](http://www.ncbi.nlm.nih.gov/nuccore/NM_015719?report=genbank) | COL5A3 | Coding | 1 | -0.408 | -0.251 | 152 |
| [NM_001004713](http://www.ncbi.nlm.nih.gov/nuccore/NM_001004713?report=genbank) | OR1I1 | Coding | 1 | -0.408 | -0.256 | 151 |
| [NM_001166305](http://www.ncbi.nlm.nih.gov/nuccore/NM_001166305?report=genbank) | TMEM44 | Coding | 1 | -0.404 | -0.239 | 165 |

***:** only the top 30 target genes are listed.

Seqname: the name of the sequence.

GeneSymbol: the official gene symbol of the sequence.

Type: the type of the transcript.

Total: the total number of the binding sites on the targets.

Context+: the sum of the context+ scores used in TargetScan after version 6.0, more negative is better.

Context: the sum of the context scores used in TargetScan before version 5.x, more negative is better. Structure: the sum of the structure scores used in miRanda, the higher is better.

Energy, the sum of the free energy predicted by miRanda, more negative is better.

**Supplemental Figures**

**
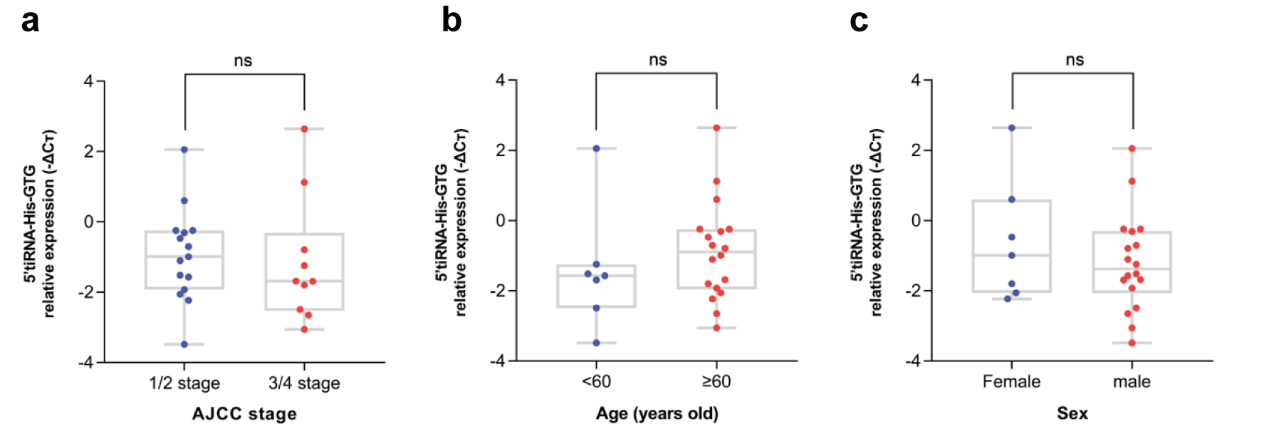
**

**Figure S1. Distribution of the 5’tiRNA-His-GTG relative expression level in different groups.** **(a)** The expression level of 5’tiRNA-His-GTG showed no significant difference between early stage and advanced stage CRC. **(b)** There was no difference in the expression level of 5’tiRNA-His-GTG above and below the age of 60 years old. **(c)** The expression level of 5’tiRNA-His-GTG was not significantly different between males and females. ns: not significant. CRC, colorectal cancer.


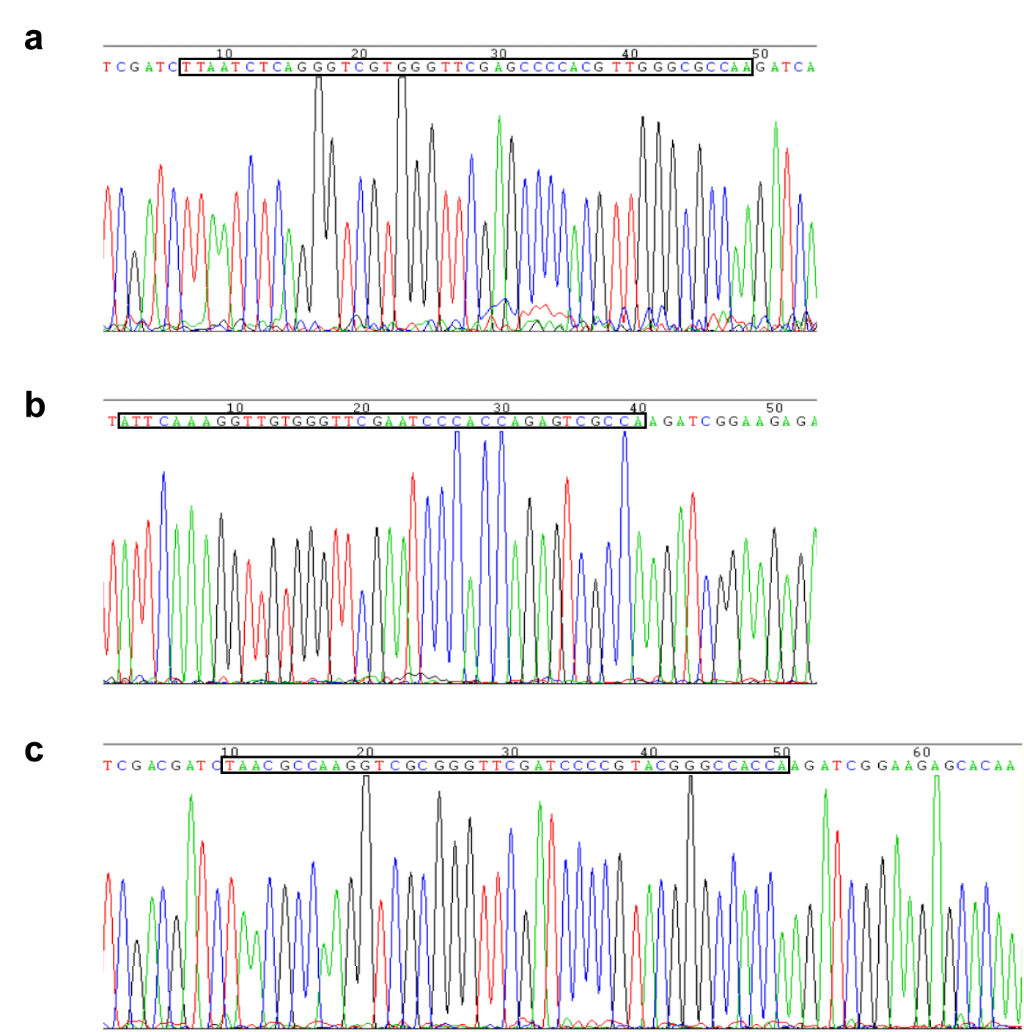


**Figure S2. Sanger sequencing of the PCR products of qRT-PCR. (a)** 3’tiRNA-Lys-CTT. **(b)** 3’tiRNA-Arg-TCT-2-1. **(c)** 3’tiRNA-Ile-AAT.


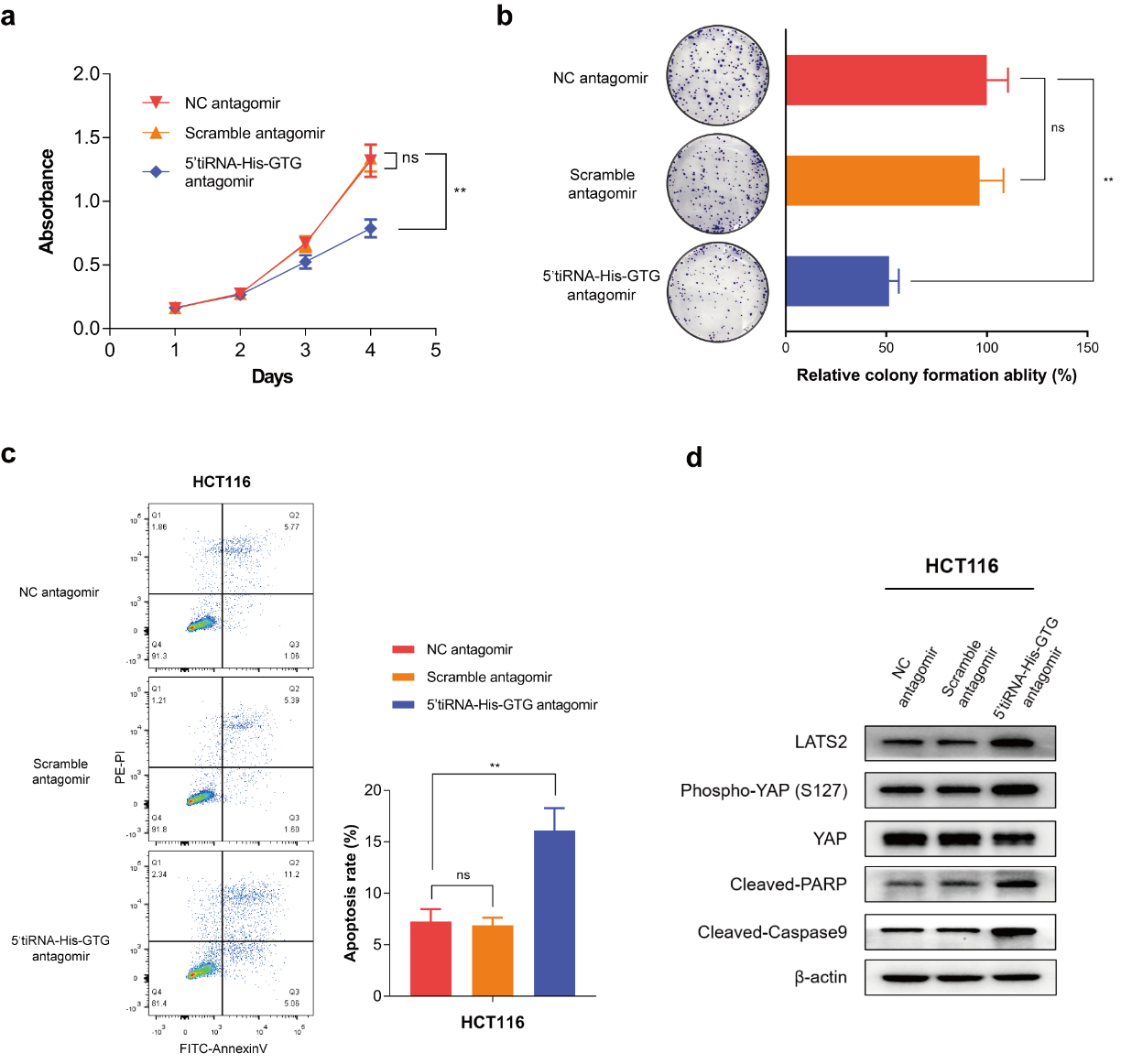


**Figure S3. The 5’tiRNA-His-GTG antagomir performs the same function as 5’tiRNA-His-GTG inhibitor in HCT116 cells.** **(a)** The 5’tiRNA-His-GTG antagomir inhibited cell proliferation. **(b)** The 5’tiRNA-His-GTG antagomir reduced colony formation ability. **(c)** The 5’tiRNA-His-GTG antagomir increased the cell apoptosis rate. **(d)** The results of western blotting showed that the 5’tiRNA-His-GTG antagomir obviously increased the level of LATS2, phospho-YAP (Ser127), cleaved-PARP, and cleaved-caspase 9, but decreased the level of YAP. **p* < 0.05, ***p* < 0.01, ns: not significant. All the data are representative of at least three independent experiments and presented as the means ± SD. qRT-PCR, quantitative real-time reverse transcription PCR; LATS2, large tumor suppressor kinase 2; YAP; Yes-associated protein; PARP, poly (ADP-Ribose) polymerase 1.


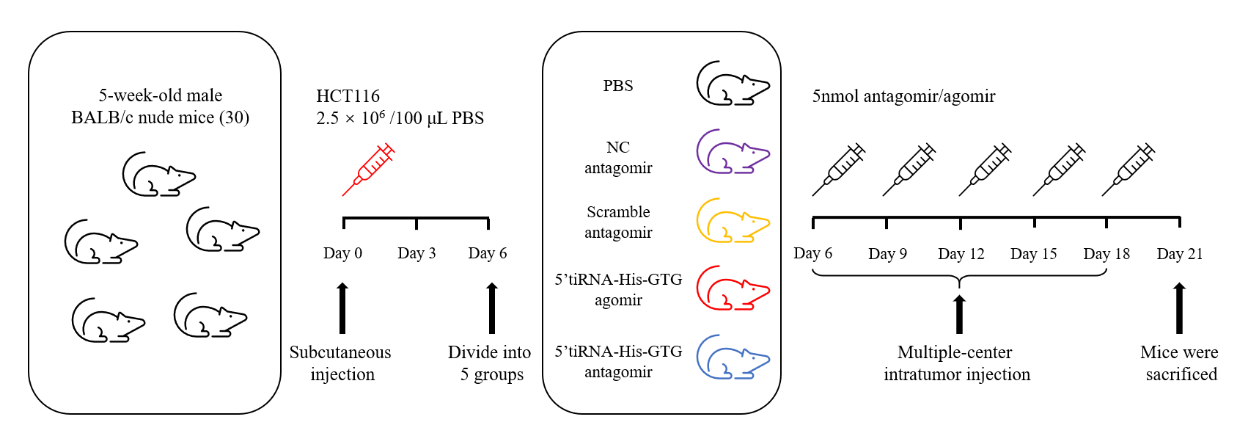


**Figure S4. Schematic diagram of animal experiments.** HCT116 cells (2.5 × 10^6^) were injected subcutaneously into the right axilla of each mouse (5-week-old male BALB/c nude mice) to establish the CRC xenograft model. 6 days after subcutaneous inoculation, mice were randomly divided into different groups for different treatments and finally sacrificed for tumor assessment.


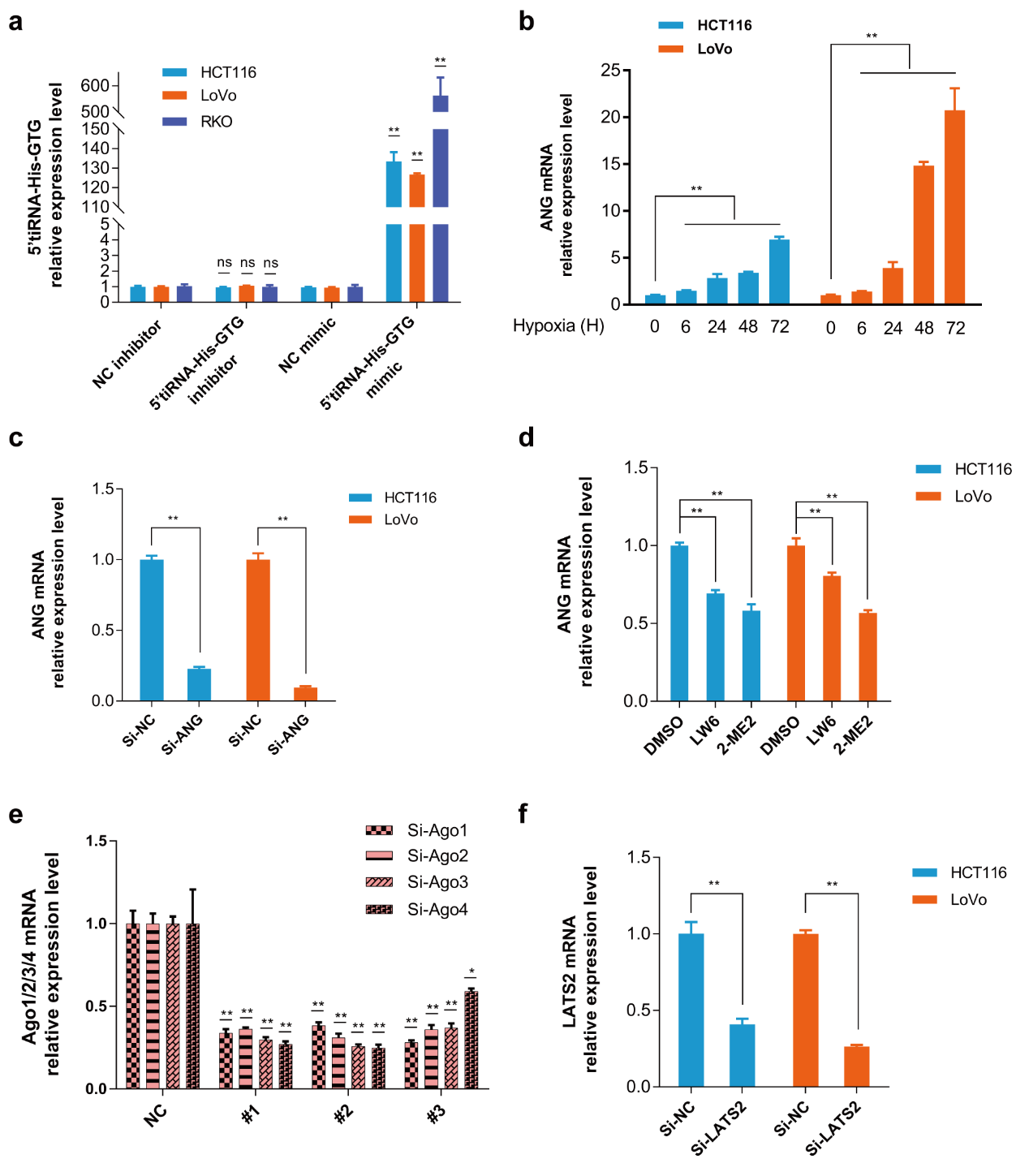


**Figure S5. qRT-PCR validation of different genes.** (**a)** The expression level of 5’tiRNA-His-GTG in the cells after transfection of the 5’tiRNA-His-GTG mimic and inhibitor. (**b)** Relative expression level of *ANG* is upregulated under hypoxic conditions. **(c)** The knockdown effect of Si-ANG was evaluated under hypoxic conditions (48H). **(d)** *ANG* mRNA expression is downregulated using a HIF-1α inhibitor under hypoxic conditions (48H). **(e)** The knockdown effect of Si-AGO1/2/3/4 was evaluated. (**f)** The knockdown effect of Si-LATS2 was evaluated. **p* < 0.05, ***p* < 0.01, ns: not significant. All the data are representative of at least three independent experiments and presented as the means ± SD. qRT-PCR, quantitative real-time reverse transcription PCR; ANG, angiogenin; HIF-1α, hypoxia inducible factor 1 subunit alpha; AGO, argonaute; LATS2, large tumor suppressor kinase 2.


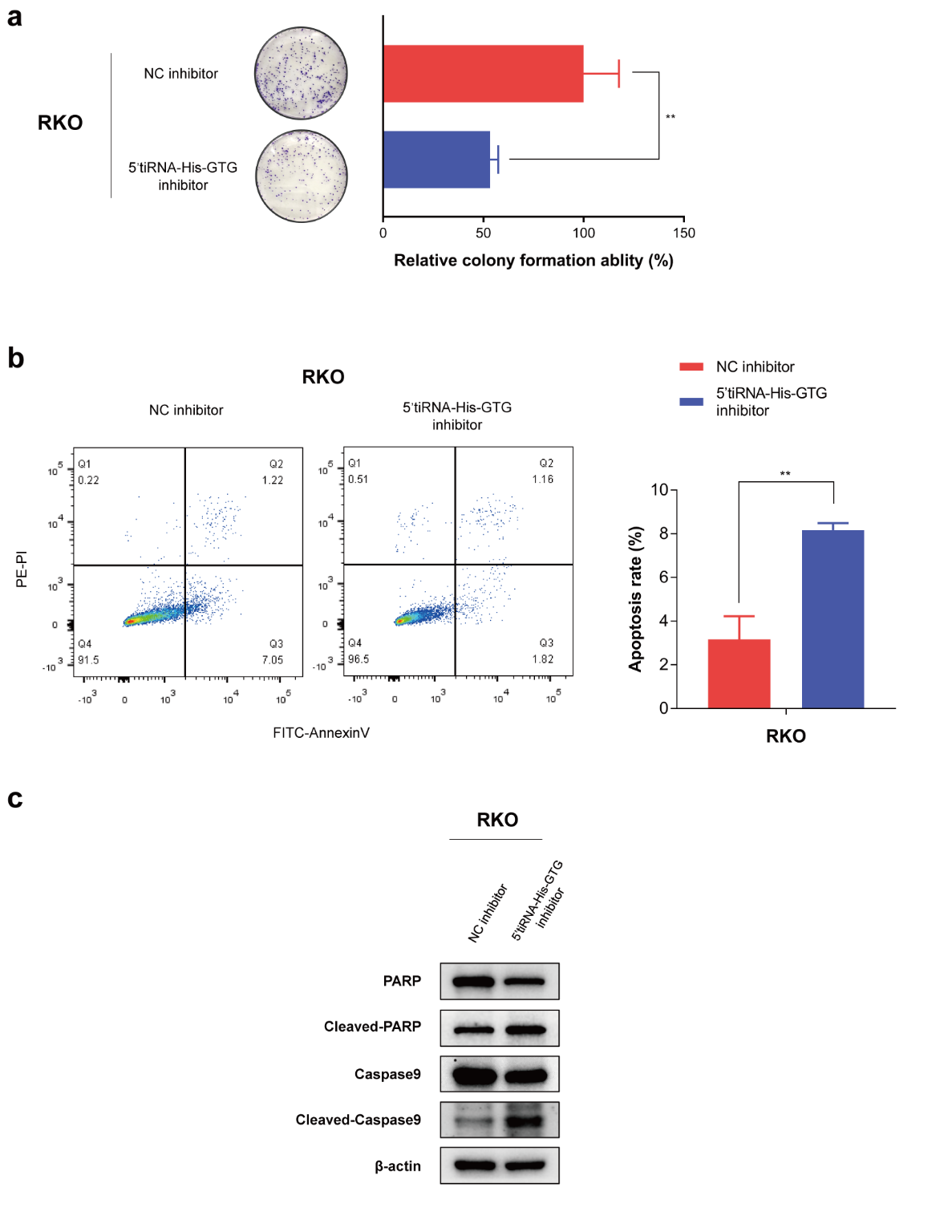


**Figure S6. 5’tiRNA-His-GTG inhibition reduces anchorage-dependent growth and induces apoptosis in RKO cells. (a)** Inhibition of 5’tiRNA-His-GTG reduced colony formation of RKO cells. **(b)** Flow cytometry analysis revealed that 5’tiRNA-His-GTG inhibitor could induce cell apoptosis. **(c)** The levels of apoptosis indicators (PARP, Cleaved-PARP, Caspase9, Cleaved-Caspase9) were detected using western blotting. **p* < 0.05, ***p* < 0.01. All data are presented as the means ± SD. PARP, poly (ADP-Ribose) polymerase 1.


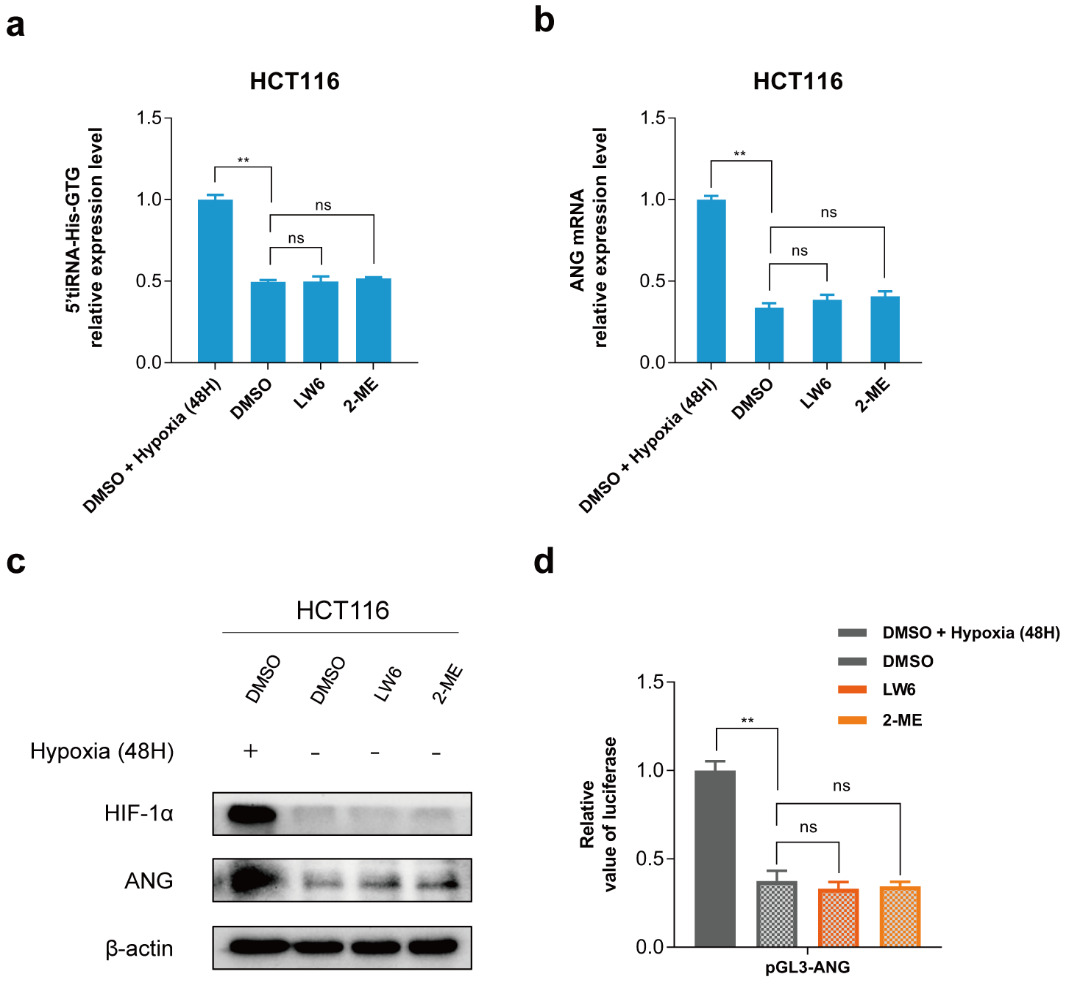


**Figure S7. LW6 and 2-ME have no effect on** **HIF-1α/ANG/5’tiRNA-His-GTG axis under normoxia in HCT116 cells.** **(a)** Under normoxia**,** the expression level of 5’tiRNA-His-GTG remains unchanged with the treatment of LW6 or 2-ME. **(b)** Under normoxia**,** the expression level of *ANG* mRNA remains unchanged with the treatment of LW6 or 2-ME. **(c)** The results of western blotting showing that LW6 or 2-ME could not affect the level of HIF-1α and ANG under normoxic environment. **(d)** Luciferase reporter assay reveals that LW6 or 2-ME has no regulating effect on *ANG* promotor under normoxic condition. The DMSO + Hypoxia (48H) group is considered as a positive control. **p* < 0.05, ***p* < 0.01, ns: not significant. All data are presented as the means ± SD. ANG, angiogenin; HIF-1α, hypoxia inducible factor 1 subunit alpha.


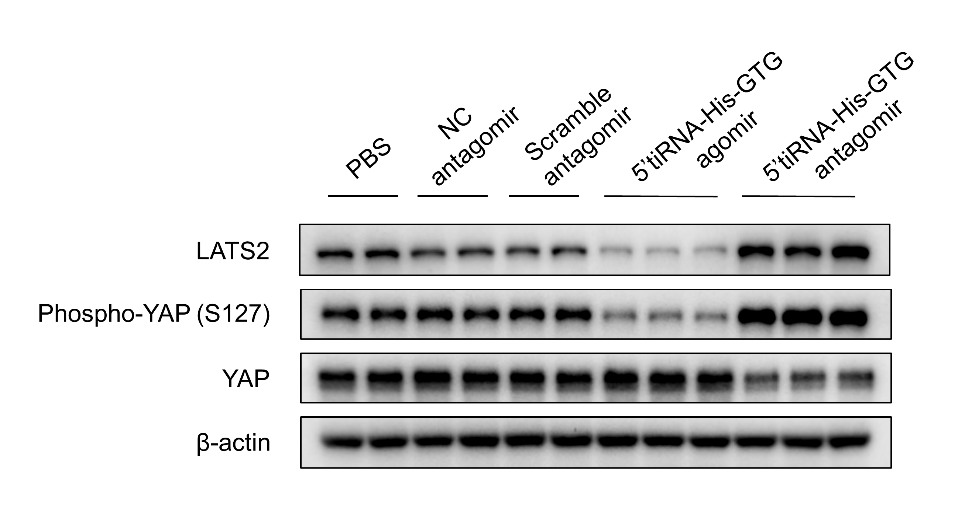


**Figure S8. The expression level of LATS2, phospho-YAP (Ser127), and YAP in the xenograft tumors.** *In vivo* experiment in which treatment using the 5’tiRNA-His-GTG agomir decreased the expression of LATS2 and phospho-YAP (Ser127), while increased the expression of YAP in isolated tumors. Two independent and representative samples are shown for control groups (PBS, NC antagomir, Scramble antagomir), Three independent and representative samples are shown for experimental groups (5’tiRNA-His-GTG agomir and 5’tiRNA-His-GTG antagomir). LATS2, large tumor suppressor kinase 2; YAP: yes-associated protein.


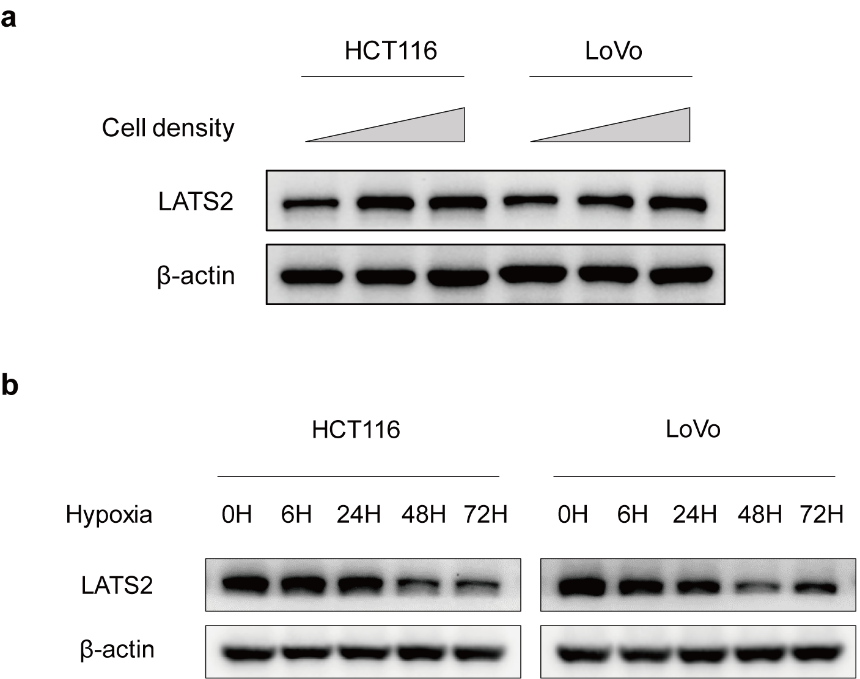


**Figure S9.** **The expression level of LATS2 upon different cell densities and various hypoxia time.** **(a)** A high cell density increased the expression of LATS2 protein. **(b)** The expression of LATS2 protein decreased as hypoxia was prolonged. LATS2, large tumor suppressor kinase 2.


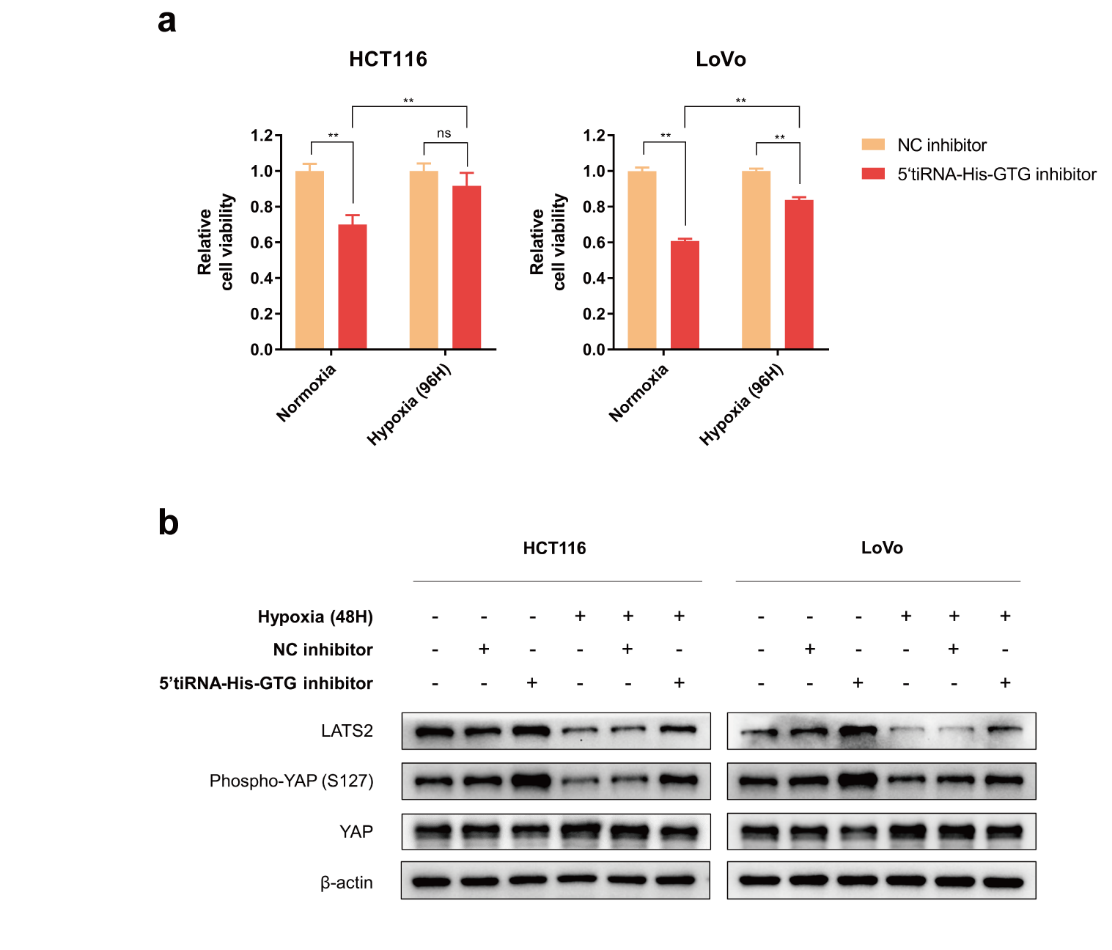


**Figure S10. The role of 5’tiRNA-His-GTG inhibitor under hypoxic environment.** **(a)** CCK8 assay showing 5’tiRNA-His-GTG inhibitor suppresses the cell viability, while hypoxic treatment (96H) rescues the cell viability in HCT116 and LoVo cells. **(b)** 5’tiRNA-His-GTG inhibitor attenuated hypoxia-induced YAP activation in HCT116 and LoVo cells. **p* < 0.05, ***p* < 0.01, ns: not significant. All data are presented as the means ± SD. LATS2, large tumor suppressor kinase 2; YAP: yes-associated protein.


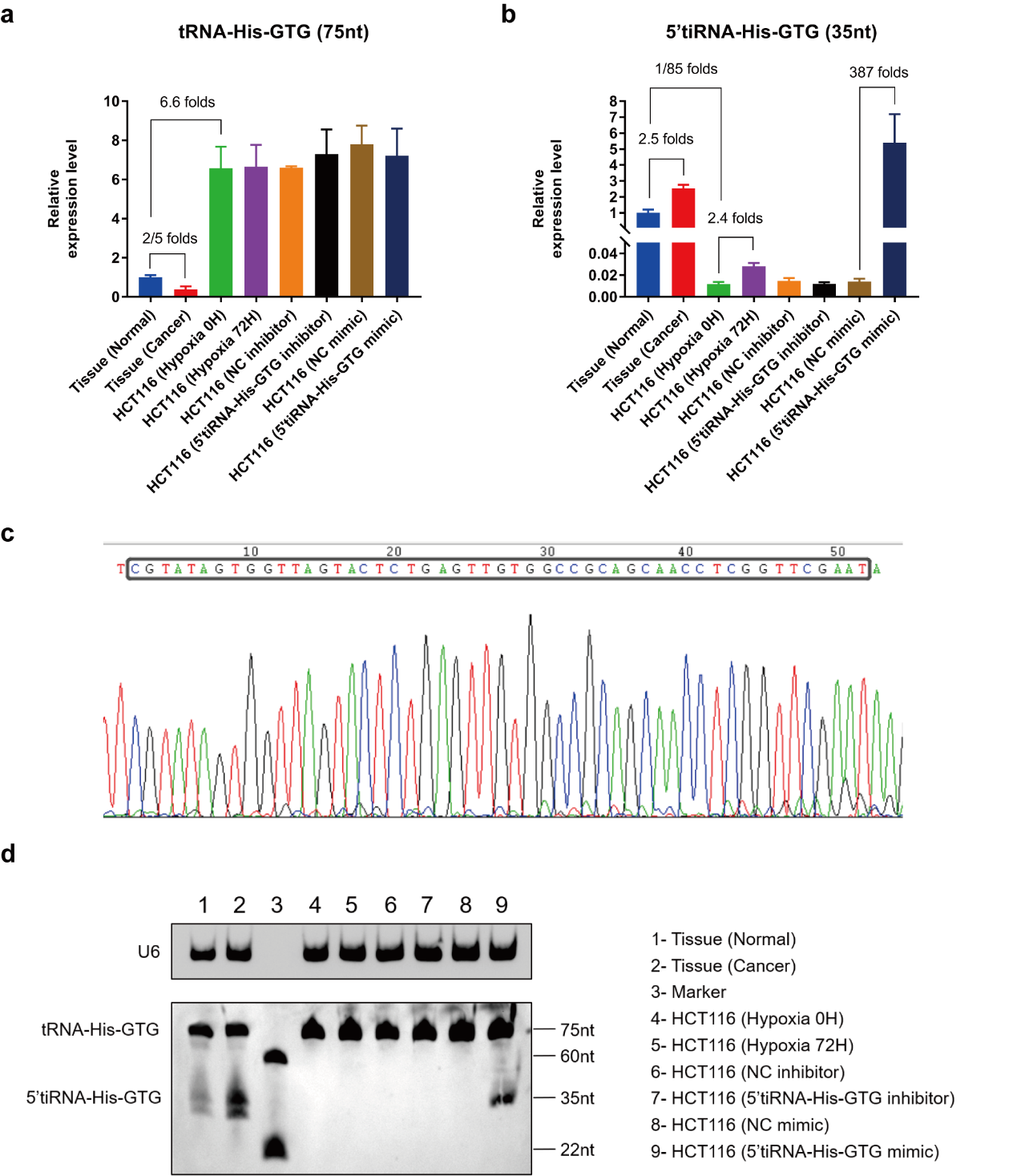


**Figure S11. The expression level of tRNA-His-GTG and 5’tiRNA-His-GTG in various groups using qRT-PCR and northern blot. (a)** The expression level of tRNA-His-GTG in various groups using qRT-PCR. **(b)** The expression level of 5’tiRNA-His-GTG in various groups using qRT-PCR (Stem-loop RT Method without RNA pre-treatment). **(c)** The product of qRT-PCR for tRNA-His-GTG was confirmed by Sanger sequencing **(d)** The expression level of tRNA-His-GTG and 5’tiRNA-His-GTG in various groups using northern blot. All the data are representative of at least three independent experiments and presented as the means ± SD.
